# Anisotropic Thermal Conductivity in Imine-Linked Two-Dimensional Polymer Films Produced by Interfacial Polymerization

**DOI:** 10.1021/acsnano.4c17126

**Published:** 2025-05-14

**Authors:** Yuxing Liang, Kiana A. Treaster, Ayan Majumder, Manoj Settipalli, Kanishka Panda, Shravan Godse, Rupam Roy, Ratul Mali, Zhongyong Wang, Yuxuan Luan, Peijie Hu, Keith Searles, David C. McLeod, Kirt A. Page, Dayanni Bhagwandin, Edgar Meyhofer, Pramod Reddy, Alan J. H. McGaughey, Austin M. Evans, Jonathan A. Malen

**Affiliations:** 1 Department of Mechanical Engineering, 6612 Carnegie Mellon University, 5000 Forbes Ave, Pittsburgh, Pennsylvania 15213, United States; 2 George and Josephine Butler Polymer Research Laboratory, Department of Chemistry, Center for Macromolecular Science and Engineering, 3463 University of Florida, Gainesville, Florida 32611, United States; 3 Department of Materials Science and Engineering, 3463 University of Florida, Gainesville, Florida 32611, United States; 4 Center for Catalysis, Department of Chemistry, 3463 University of Florida, Gainesville, Florida 32611, United States; 5 Department of Mechanical Engineering, 2370 G.G. Brown Laboratory, 1259 University of Michigan, 2350 Hayward, Ann Arbor, Michigan 48109, United States; 6 DEVCOM Army Research Laboratory, Aberdeen Proving Ground, Maryland 21005, United States; 7 Materials and Manufacturing Directorate, Air Force Research Laboratory, WPAFB, Dayton, Ohio 45433, United States; 8 UES A BlueHalo Company, Dayton, Ohio 45432, United States

**Keywords:** two-dimensional polymers, covalent organic frameworks, anisotropic thermal conductivity, frequency domain thermoreflectance, high-resolution calorimeters, molecular dynamics simulations

## Abstract

Anisotropic thermal transport was measured in imine-linked two-dimensional polymer (2DP) films that were prepared by interfacial polymerization. Measurements of both in-plane (*k*_∥_) and cross-plane (*k*_⊥_) thermal conductivities relied on preparing free-standing 2DP films that were readily transferred for different measurement configurations. We polymerized two 2DP (Per-PDA and TAPPy-PDA) films at a liquid–liquid interface. These polycrystalline, imine-linked 2DP films are 100–200 nm in thickness and were measured by frequency domain thermoreflectance to extract *k*_⊥_ and a suspended calorimetric platform technique to evaluate *k*_∥_. We find that *k*_∥_ is larger than *k*_⊥_ in both materials at room temperature, leading to anisotropy ratios (*k*_∥_/*k*_⊥_) as high as 2.3. We attribute this behavior to the fact that the stiff, in-plane covalent bonds of 2DPs transport heat more effectively than the flexible, supramolecular cross-plane interactions. Variable–temperature measurements revealed a positive correlation between temperature and thermal conductivity, which we attribute to phonon scattering from grain boundaries and defects in the polycrystalline 2DP films. Molecular dynamics simulations of pristine crystals predict larger thermal conductivities and anisotropy ratios exceeding 7. The simulations suggest that as higher quality 2DP films become available, higher thermal conductivities and anisotropy ratios will also manifest.

## Graphical abstract

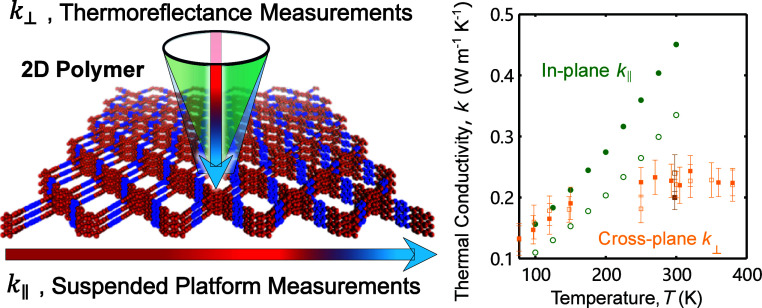

## Introduction

Two-dimensional polymers (2DPs) are planar macromolecules constructed by reacting planar multifunctional nodes and linkers that can stack through van der Waals interactions in the cross-plane direction.^1–3^ By judicious monomer selection, it is possible to create crystalline lattices with deterministically installed chemical functionality and reliably controlled pore size. The structural precision inherent to 2D polymerization,^4,5^ typically guided by reversible condensation chemistries, facilitates the construction of covalently linked layers. The topological regularity of 2DP sheets endows them with a unique combination of inherent crystallinity, permanent porosity, and reticular chemical design. This constellation of properties endows 2DPs with material properties that are often difficult to obtain in other polymeric systems. Given the anisotropy of in-plane covalent bonds and cross-plane van der Waals interactions, electrically insulating 2DPs may exhibit anisotropic thermal transport, which is valued for on-chip heat spreading in electronics.

Macromolecules are conventionally considered to be thermal insulators as quantified by thermal conductivities of 0.1–0.3 W m^–1^ K^–1^.^6–8^ Thermal conductivity relates the heat flux to the temperature gradient in matter and in nonmetals results from the cumulative contributions of vibrational energy carriers. In crystals these vibrations are known as phonons. It is understood that a high thermal conductivity (*k*) can be realized in aligned polymer chains with long-range crystallinity.^9,10^ This morphology enhances the mean free paths of the heat-carrying phonons. Typically, high thermal conductivities in organic materials are achieved by processing methods that take disordered polymers and increase their crystallinity through drawing or stretching.^11–13^ It has also been observed that noncovalent interactions that rigidify polymeric materials also increase *k.* ^14–16^ These interactions are reminiscent of the interlayer van der Waals interactions in 2DPs, which may also stiffen these materials. The inherent crystallinity and supramolecular rigidification of 2DPs could, in principle, make these materials intrinsically thermally conductive without the need for postsynthetic processing.

The anisotropic structure of 2DPs should result in anisotropic thermal conductivities. Molecular dynamic (MD) simulations predict that the stiff covalent in-plane interactions of 2DPs will lead to high in-plane thermal conductivities (>1 W m^–1^ K^–1^).^17–20^ In contrast, the weak dispersion forces that join 2DP layers into stacks should be less effective at transporting heat. The thermal conductivity of a boronate ester-linked 2DP heterostructured film (2DP/graphene/SiO_2_/Si) was recently measured by time and frequency domain thermoreflectance techniques,^21^ which revealed a *k*_⊥_ value of 1.03 ± 0.15 W m^–1^ K^–1^. Due to the solvothermal film-forming approach used in this report, it was not possible to measure *k*_∥_. Therefore, the authors estimated an anisotropy ratio (*k*_∥_/*k*_⊥_) of ∼3 using MD simulations and thus inferred that *k*_∥_ = 3.5 W m^–1^ K^–1^. These findings are consistent with MD simulations performed in a separate study,^22^ which revealed that 24 distinct 2DP systems had directionally anisotropy ratios (*k*_∥_/*k*_⊥_) > 3. However, further measurements of 2DP crystals have not been reported and this anisotropy has not yet been experimentally confirmed.

Here, we investigate the thermal properties of two distinct imine-based 2DP systems and experimentally confirm their anisotropic thermal conductivity. Previous research has established several methods for producing imine-based, crystalline, free-standing 2DP thin films.^23–25^ Their chemical stability, versatility, and well-established synthetic protocols make them an ideal material for exploring anisotropic thermal properties. Free-standing polycrystalline 2DP thin films were synthesized using interfacial polymerization (Figure 1A). Cross-plane and in-plane thermal conductivities were measured by employing thermoreflectance,^26–28^ and microfabricated suspended calorimetric platforms,^29,30^ confirming the anisotropic thermal conductivity of the imine-linked 2DPs at room temperature. To further understand the underlying heat transfer mechanisms, variable-temperature experiments were conducted and compared with the results of MD simulations. These results suggest that thermal conductivity in these 2DP films is limited by grain boundaries and defects, which indicates that higher quality samples will have higher thermal conductivities and anisotropy ratios.

**Figure 1:**
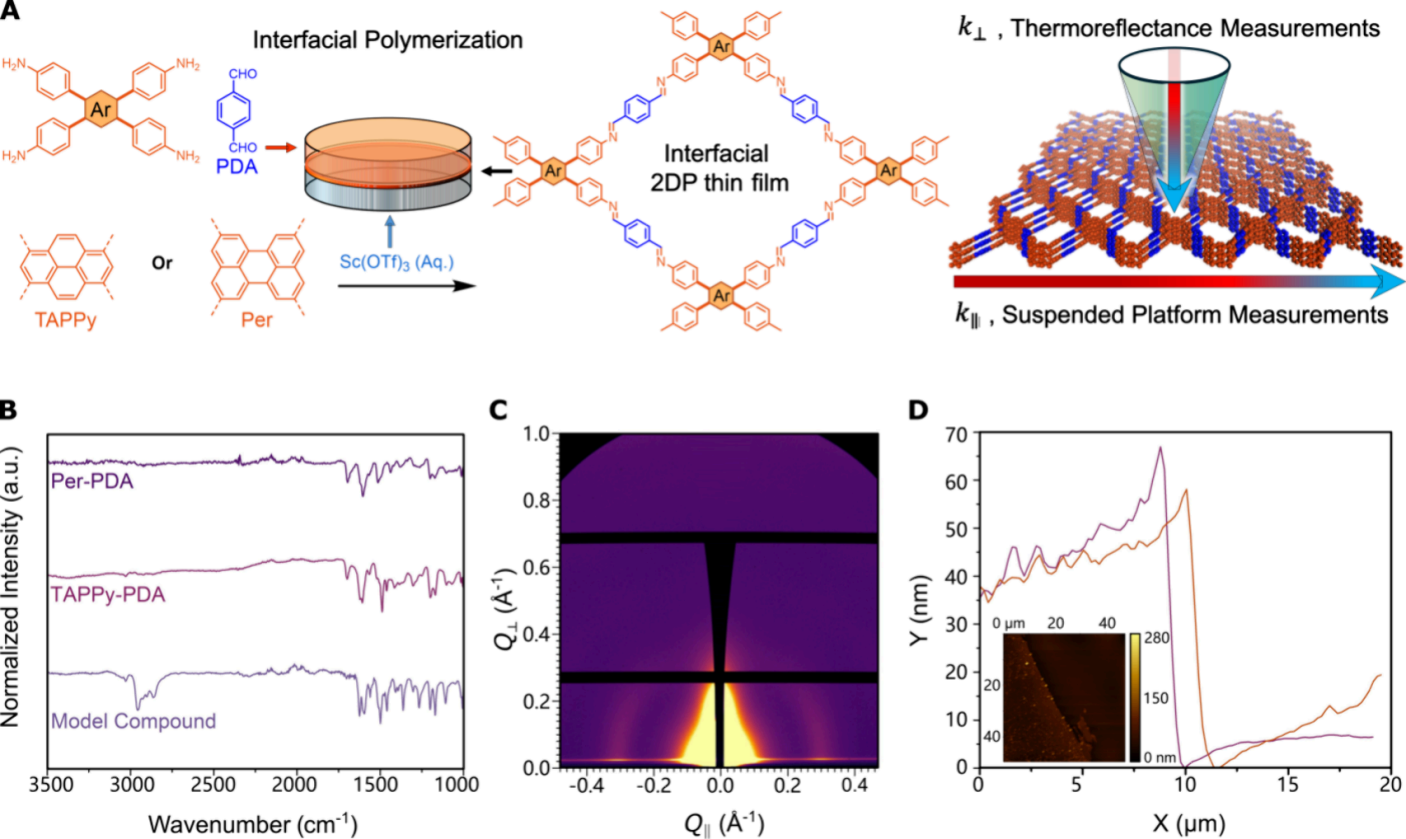
2DP Synthesis and chemostructural characterization. (A) Synthesis and characterization of imine-linked 2DPs. (B) FT-IR spectroscopy of Per-PDA, TAPPy-PDA, and the reference model compound shows an imine stretching band observed at approximately 1600 cm^–1^. (C) GIWAXS patterns of Per-PDA thin film, in-plane scattering feature at 0.3 Å^–1^. (D) Line cut of Per-PDA thin film from atomic force microscope scan (inset).

## Results and Discussion

We prepared two imine-linked free-standing 2DP films (Figure 1A). We first dissolved one of two tetra-aminophenyl functionalized nodes (1.56 mM), 1,3,6,8-tetrakis­(4-aminophenyl)­pyrene (TAPPy) and 2,5,8,11-tetrakis­(4-aminophenyl)­perylene (Per), with a difunctional phenylene dialdehyde (PDA, 3.12 mM) in a mixture of THF:mesitylene (4:1 vol:vol). We then layered this organic mixture onto an aqueous solution with a Lewis acid transimination catalyst (Sc­(OTf)_3_, 5 mM). This approach yielded homogeneous yellow thin films at the aqueous/organic interface after standing at 30 min at ambient temperature (see Section S1). Atomic force microscopy measurements (Figure 1D) performed on some of the thinnest films revealed that they were 30–40 nm thick with a root mean squared roughness of 5–15 nm. Thicker films (100–200 nm thick) synthesized by the same approach were collected for chemostructural characterization or transferred to a substrate for thermal conductivity measurements.

The layered imine-linked structure of the Per-PDA and TAPPy-PDA 2DPs was confirmed with X-ray scattering and vibrational spectroscopy measurements. Fourier-transform infrared (FT-IR) spectroscopy of thin films (Figure 1B) and polycrystalline 2DP powders prepared under homogeneous polymerization conditions confirmed the formation of imine-bonds with the appearance of an imine (−C=N) stretch at approximately 1600 cm^–1^. The appearance of imine features was commensurate with the disappearance of amine (3356 cm^–1^) and aldehyde (1700 cm^–1^) features in the monomer spectra. Grazing-incidence wide-angle X-ray scattering (GIWAXS) revealed an anisotropic scattering feature at 0.3 Å^–1^ (Figure 1C), which is consistent with the previously reported ⟨100⟩ Bragg feature of this material.^31,32^ The intensity of this feature is concentrated on the *Q*_||_ plane. This result suggests that these films are comprised of 2DP layers that are preferentially, but not perfectly, aligned parallel to the surface, which is consistent with other reports of interfacial 2D polymerization using Lewis acid catalysts.^33,34^ The low scattering intensity from organic thin films made obtaining a TAPPy-PDA GIWAXS profile challenging. However, polycrystalline powders of both Per-PDA and TAPPy-PDA produced under homogeneous reaction conditions yielded X-ray diffraction profiles with several Bragg reflections that suggest that high-quality crystallites form under these polymerization conditions. Importantly, we expect these films to be polycrystalline mosaics to which standard characterization methods are not sensitive, as observed in previous reports.^35–37^ Taken together, these measurements suggest that thin films produced by interfacial polymerization are polycrystalline and preferentially oriented. See Section S1 for additional details on structural characterization.

We employed frequency domain thermoreflectance (FDTR), a noncontact laser-based technique, to measure *k*_⊥_ of the 2DPs (Figure 2A; see Section S2). FDTR uses a modulated pump laser (488 nm) that periodically heats the Au transducer that is evaporated on top of the 2DP. A probe laser (532 nm) is used to measure the temperature-dependent reflectance (i.e., thermoreflectance) of the Au coating. The phase lag of the temperature response with respect to the heat flux is measured for a range of modulation frequencies (Figure 2B). The phase data is then fit to an analytical solution^28^ of the heat diffusion equation for periodic laser heating of a layered solid to determine the unknown thermal conductivity of the 2DP. FDTR is sensitive to *k*_⊥_ and not to *k*_∥_ in this configuration because heat flows normal to the thin film into the sapphire substrate (see Section S2). The heat capacity of the 2DP required for fitting the phase data was determined separately using harmonic lattice dynamics calculations. This approach yielded reasonable agreement with a previously measured 2DP heat capacity for COF-5 (see Section S4).^21^

**Figure 2:**
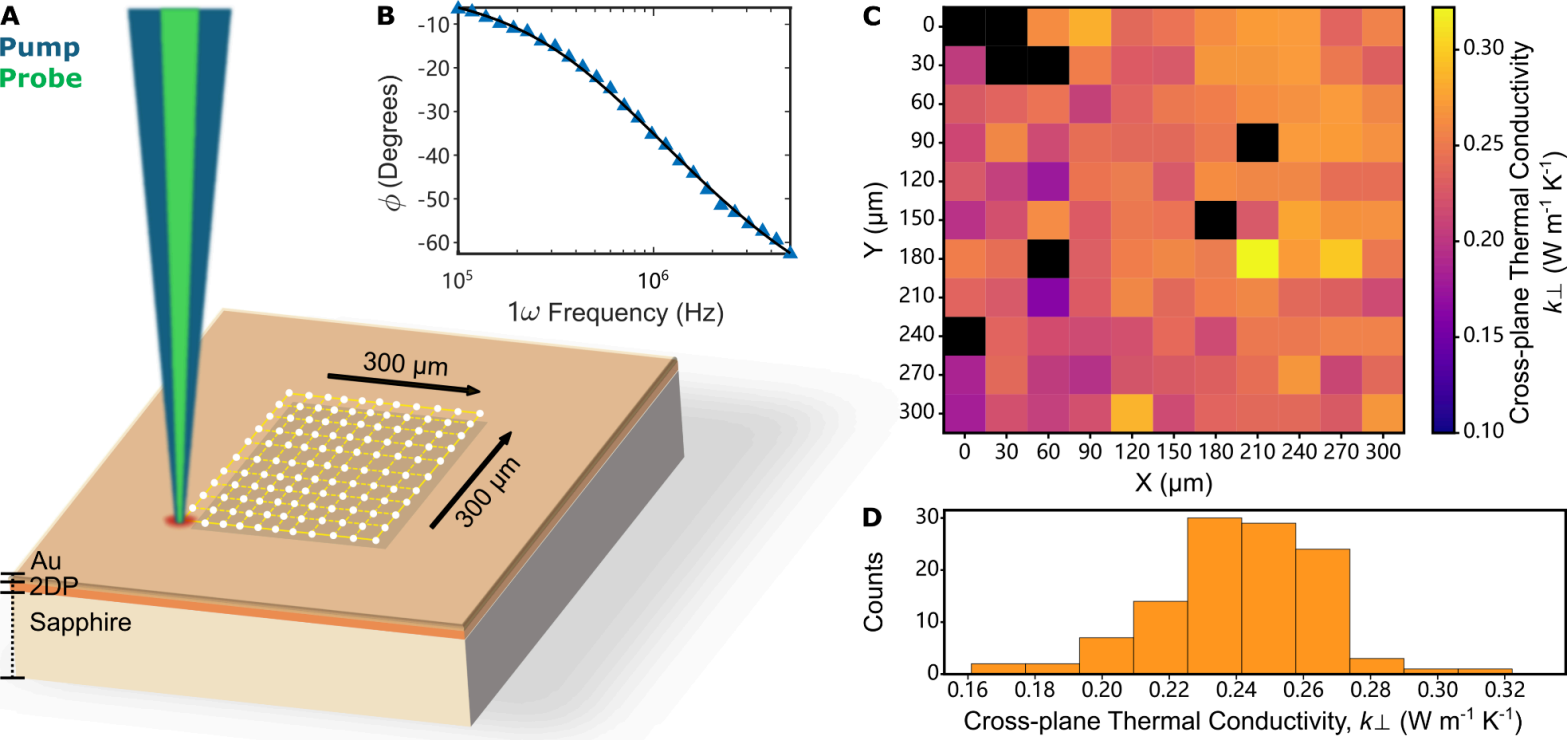
Cross-plane thermal conductivity measurement using FDTR. (A) Schematic illustrating how lateral scanning FDTR measurements are performed. (B) Measured phase lag of temperature to periodic heating (blue triangles) from an individual FDTR measurement on a Au/Per-PDA/sapphire sample and analytical fit (black solid line). (C) Heatmap of cross-plane thermal conductivities for Per-PDA film from an FDTR scan over a 300 μm × 300 μm grid. The black squares are the filtered measurements with unsatisfactory fits (see Section S2.2). (D) Histogram summarizing the cross-plane thermal conductivities from 113 individual measurements on a single Au/Per-PDA/sapphire sample.

To prepare our samples for FDTR, we lifted 2DP films from the liquid–liquid interface, transferred them onto an Al_2_O_3_ wafer, and then coated it with 78 ± 2 nm of Au by e-beam evaporation. We then performed profilometry on these samples and obtained thicknesses of 124 ± 2 nm for Per-PDA and 164 ± 31 nm for TAPPy-PDA (see Section S2). Previous thermoreflectance experiments performed on 2DP samples relied on a single measurement to determine *k*. Given the uncertainty in film thickness, roughness, chemical heterogeneity, and the polycrystalline nature of 2DP films, there is an uncertainty in the final extracted *k*_⊥_ value. To quantify the variability we conducted a scan with an 11 × 11 grid of FDTR measurements that were evenly spaced within a 300 μm × 300 μm square with a laser spot size of 3.4 ± 0.2 μm (Figure 2A). These measurements result in an array of 121 FDTR measurements across the 2DP film (Figure 2C). Histograms (Figure 2D for Per-PDA) summarize these distributions and we find that the mean and 90/10 confidence intervals of the cross-plane thermal conductivities for Per-PDA and TAPPy-PDA are *k*_⊥_ = 0.24 ± _0.03_^0.03^ W m^–1^K^–1^ and *k*_⊥_ = 0.20 ± _0.03_^0.02^ W m^–1^K^–1^ at room temperature. These relatively narrow distributions of thermal conductivities suggest that the films are homogeneous across the measured area, with variances arising from grain boundaries or local orientational differences of the crystallites in the films.

In-plane thermal conductivity measurements were performed using a silicon nitride (SiN) based suspended calorimetric platform (Figure 3A) that has been successfully employed in past studies of nanoscale thermal transport.^29,30,38^ The SiN calorimetric platforms consist of two suspended, coplanar membranes that were each patterned with a platinum resistor that can act as both a heater and a temperature sensor. The 2DP thin film was then carefully transferred onto the calorimeters using a micromanipulator under a stereo microscope and cut into a uniform, rectangular shape by a focused-ion beam (FIB). Subsequently, Pt was deposited at a spatial location where the 2DP films contact the SiN device to reduce the thermal boundary resistance (see Figure 3A). Control experiments (see Section 3.4 of the Supporting Information) showed that the thermal contact resistance between the 2DP sample and the devices is negligibly small even without the deposition of this Pt film, which is not surprising as the contact area between the 2DP film and the membrane is quite large (see Figure 3A).

**Figure 3:**
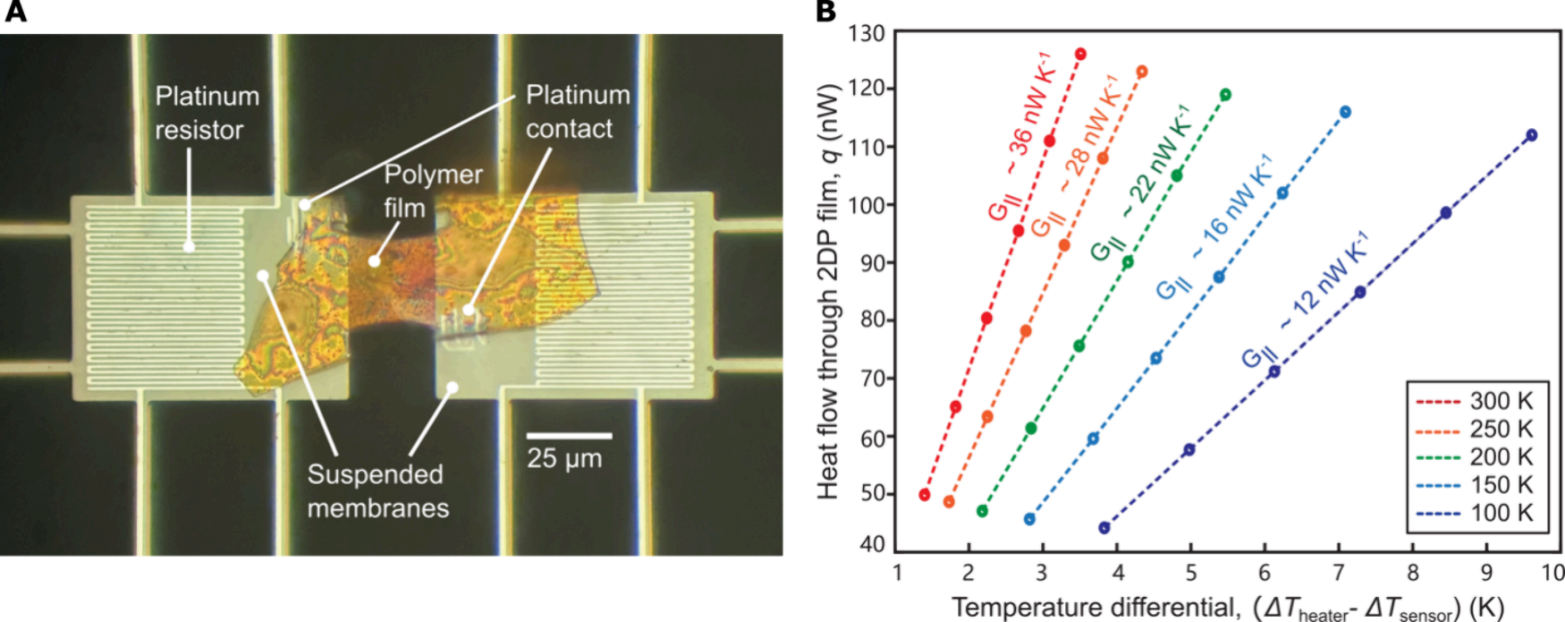
In-plane thermal conductivity measurement using suspended calorimeters. (A) Optical microscopy image of a suspended calorimeter with a transferred Per-PDA film and Pt deposition. (B) Data corresponding to a temperature-dependent (100–300 K) in-plane thermal conductance measurement of a Per-PDA film. The Per-PDA film used in this experiment was 25.0 μm long, 21.3 μm wide, and 126 nm thick. Measurements on samples with different geometries confirm that radiative losses and contact resistances do not significantly impact these measurements. These dimensions and the measured thermal conductances (*G*_||_) were used to obtain the temperature-dependent in-plane thermal conductivity (*k*_∥_).

To measure the in-plane thermal conductance (*G*_||_) of the 2DP sample, we applied a sinusoidal current of a known amplitude to the heater membrane. This current periodically modulated the temperature of the membrane (Δ*T*_heater_) (see Section S3.1). The temperature oscillation of the sensing membrane (Δ*T*_sensor_), which arises from the heat current through the suspended 2DP is determined by driving a DC current of known magnitude through the Pt sensor integrated into the sensing membrane, and measuring the periodic electrical resistance change that results from its temperature oscillation (see Section S3.1). Once the temperature rise on both membranes has been determined, *G*_||_ can be estimated based on the thermal model of the system (Figure 3B and Section S3.3). The length (*l*), width (*w*), and thickness (*t*) of the film were measured with a confocal microscope (Olympus OLS 4000 LEXT). Thus, *k*_∥_ can be related to *G*_||_ as

$$k_{∥}=\frac{l\times G_{∥}}{w\times t}$$ 1

We performed these measurements on several different 2DP samples to understand the variability across samples. The average in-plane thermal conductivities of TAPPy-PDA (8 samples) and Per-PDA (6 samples) were found to be 0.46 ± 0.06 W m^–1^K^–1^ and 0.37 ± 0.04 W m^–1^K^–1^, respectively, at 300 K, where the reported uncertainty is the standard deviation across multiple samples. Variations of *k*_∥_ between samples of the same material are potentially related to small microstructural differences (grain boundaries, crystallite sizes, nonidealized orientation). The measured values of *k*_∥_ (at 300 K) are higher than the values of *k*_⊥_, corresponding to anisotropy ratios of 2.3 and 1.5 for TAPPy-PDA and Per-PDA, respectively. This measured anisotropy is consistent with the hypothesis that heat is transported more efficiently along the stiff covalent bonds within the layers than through the weaker van der Waals bonds between layers.

To gain deeper insight into the heat transfer mechanisms, we performed variable-temperature measurements of *k*_⊥_and *k*_∥_ from 77 to 400 K. For FDTR measurements, the 2DP samples were positioned within a cryostat evacuated to 10^–4^ Torr, with the lasers directed onto the sample through the cryostat window. Temperature-dependent FDTR measurements were made at a single spatial position on the films. For the high-resolution calorimeter experiments, the entire 2DP suspended platform device was placed inside a cryostat and evacuated to a pressure of 10^–6^ Torr. In both setups, the cryostat temperature was regulated using a combination of liquid nitrogen and a PID-controlled resistive heater that together provide precise temperature control.

As shown in Figure 5A, TAPPy-PDA and Per-PDA both display higher *k*_∥_ compared to their *k*_⊥_values at higher temperatures. As the temperature is decreased, the magnitude of this anisotropy decreases, particularly for Per-PDA. As the temperature increases, both *k*_⊥_and *k*_∥_ rise, which contrasts with the typical behavior of crystalline nonmetallic materials, where Umklapp scattering of phonons conventionally causes the thermal conductivity to decrease after a peak at low temperature. We hypothesize that the phonon mean free paths are limited by temperature-independent phonon scattering processes at polycrystalline grain boundaries rather than temperature-dependent Umklapp scattering. Thus, the temperature dependence of thermal conductivity in these 2DPs is primarily driven by changes in the heat capacity. For temperatures above 250 K *k*_⊥_ of both 2DPs are nearly constant, while *k*_∥_ continue to increase. This observation suggests that the phonons that carry heat in the cross-plane direction are fully activated at lower temperatures than the phonons that carry heat in the in-plane direction, consistent with prior observations in 2D layered materials. For example, in graphite *k*_⊥_ peaks at approximately 30 K, while *k*_∥_ does not peak until above 100 K.^39^

Molecular dynamics simulations of TAPPy-PDA and Per-PDA were conducted using LAMMPS^40^ to help interpret the experimental thermal conductivity observations. The atomic interactions were modeled with the universal force field (UFF)^41^ with partial charges (Figures 4C,F) computed using the extended charge equilibration (EQeq) method.^42^ A 12.5 Å cutoff was applied for the Lennard-Jones (LJ) and Coulombic interactions, with the long-range Coulombic interactions handled using the particle–particle particle-mesh method. Supercells built from 2 × 2 × 10 unit cells were found to be sufficient to remove size effects on the thermal conductivity, which was calculated using the Green–Kubo (GK) method.

**Figure 4:**
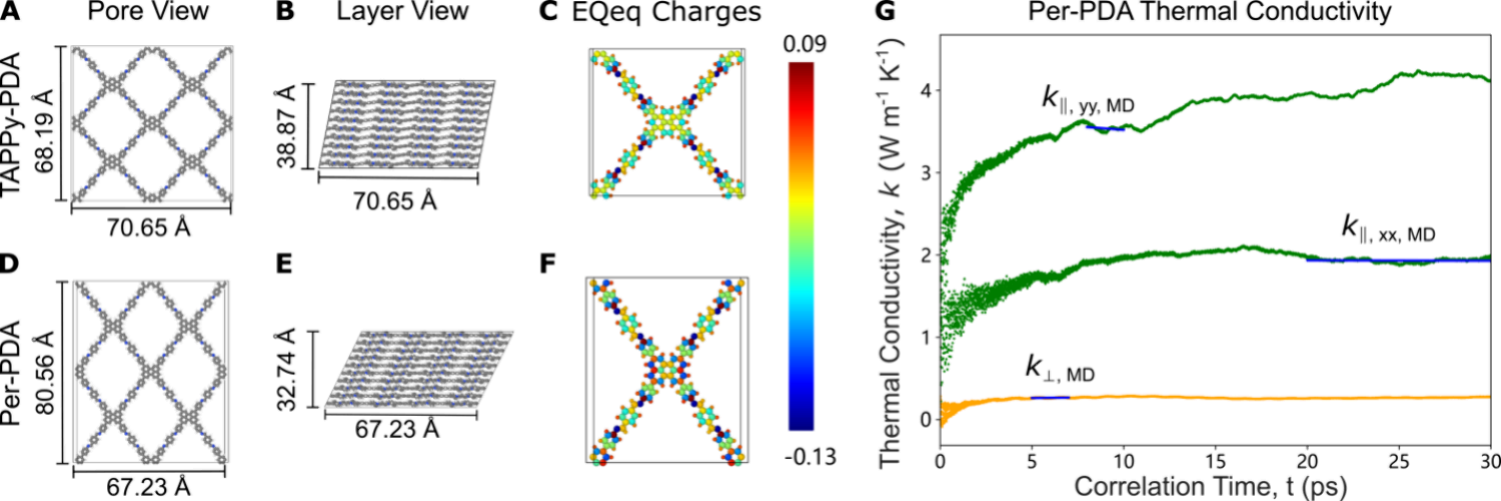
Atomistic modeling. (A, B, D, E) Relaxed TAPPy-PDA and Per-PDA structures viewed along the pore axis and showing the layer stacking. (C, F) Atomic charges calculated by the EQeq method. (G) Scaled integral of the heat current autocorrelation function for Per-PDA showing the three components of the thermal conductivity tensor. The horizontal blue lines denote the convergence region identified by an adaptive averaging technique.

The structures were equilibrated in two stages: first, in the *NVT* ensemble (i.e., constant number of atoms, volume, and temperature) for 50 ps to relax the initial structure and second, in the *NPT* ensemble (constant number of atoms, pressure, and temperature) for 1 ns to determine the unit cell parameters (lattice constants and angles). During the *NPT* simulations, the in-plane angle was constrained to 90 deg for symmetry purposes and the unit cell parameters were averaged over the final 0.4 ns. The equilibrated unit cell parameters were then applied in simulations for calculating thermal conductivity (Figures 4A,B,D,E). This approach ensures that structural fluctuations do not create nonphysical stress in the Green–Kubo calculations. After equilibration through 50 ps of *NVT* simulation, the heat flux was calculated in a further 500 ps of *NVE* (i.e., constant number of atoms, volume and energy) simulation. The heat flux autocorrelation function was then computed and results were averaged over 15 initial velocity conditions to extract the thermal conductivity tensor components *k*_∥, xx, MD_, *k*_∥, yy, MD_, and *k*_⊥, MD_ using an adaptive averaging technique (Figure 4G), to minimize the effects of random noise.^43^ For TAPPy-PDA, minimal anisotropy is expected in the in-plane direction, so that the averaged value of *k*_∥, xx, MD_ and *k*_∥, yy, MD_ is reported as *k*_⊥, MD_ in Figure 5B.

**Figure 5:**
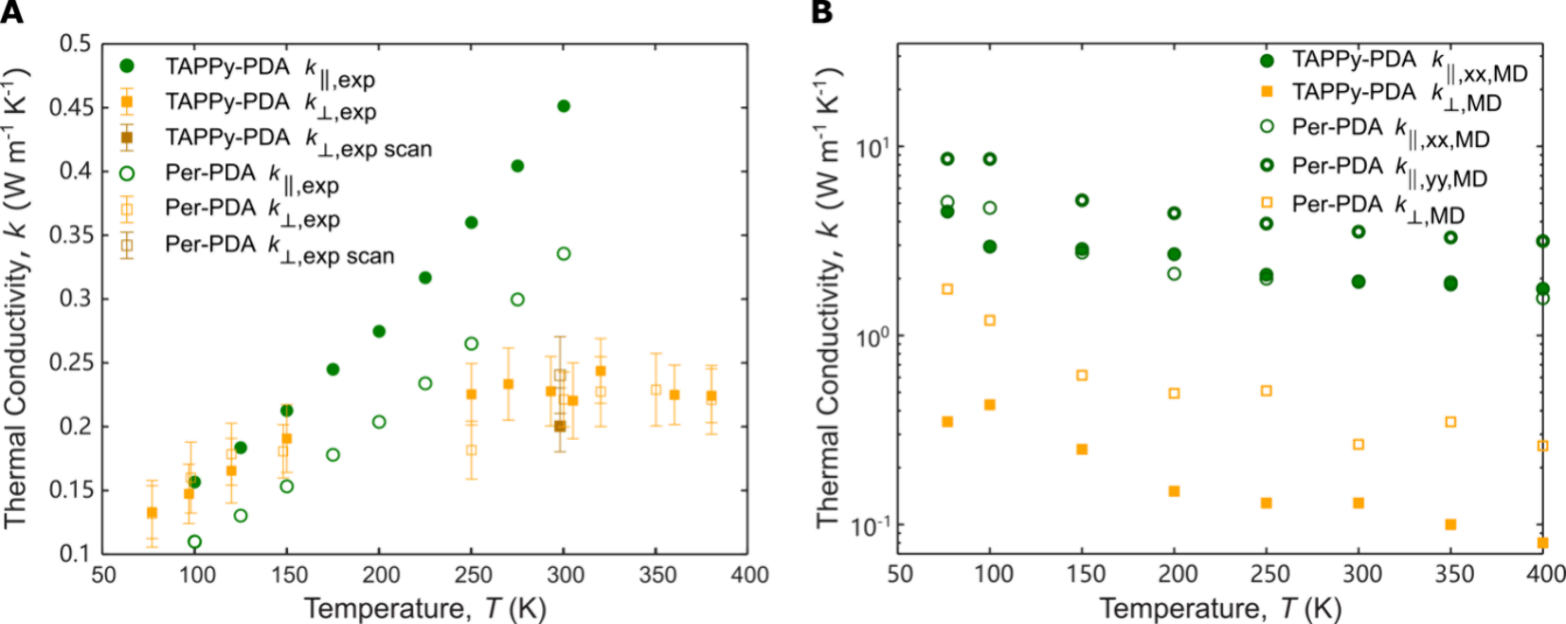
Temperature-dependent thermal conductivity of TAPPy-PDA and Per-PDA. (A) Experimental *k*_∥, exp_ and *k*_⊥, exp_ for both TAPPy-PDA and Per-PDA increase as temperature increases. Data points from room temperature spatial FDTR scans are shown as *k*_⊥, exp scan_. (B) MD predictions of *k*_∥, MD_ and *k*_⊥, MD_ instead decrease with temperature. Because Per-PDA exhibits an in-plane anisotropy, *k*_∥, xx, MD_ and *k*_∥, yy, MD_ are shown separately (though this was not resolved experimentally in the polycrystalline samples). MD simulations show a higher anisotropy in thermal conductivity compared to experimental results. We hypothesize that these discrepancies between experiments and MD simulations are due to defects and misorientation of crystallites in the experimental samples and/or the classical nature of MD simulations.

As shown in Figure 5B, the MD-predicted in-plane thermal conductivities for TAPPy-PDA and Per-PDA are larger than the experimentally measured values at all temperatures. Furthermore, the MD in-plane thermal conductivities decrease with increasing temperature, which opposes the experimental trends. There are two potential origins of these discrepancies. First, MD simulations are classical, such that all vibrational modes are activated, leading to a larger, temperature-independent heat capacity than in the experiments and thus the potential for a larger thermal conductivity. Second, the MD simulations are performed on perfect, oriented crystals and thus do not account for phonon scattering at grain boundaries and defects, or layer misorientation/nonideal stacking. Thermal conductivity will be larger in the absence of these effects and will be instead controlled by phonon–phonon scattering, which increases with increasing temperature, leading to the predicted decreasing trend. Including defects in the MD structures is expected to reduce the discrepancies. However, running simulations with grain boundaries, the most likely source of additional phonon scattering, would require prohibitively large systems. For example, to model 4 grains of size 50 nm would require more than 20 million atoms, making it computationally intractable.

In the cross-plane direction, the MD-predicted thermal conductivities also decrease with increasing temperature in contrast to the weak temperature dependence in the measurements. For TAPPy-PDA, the magnitudes of the predicted thermal conductivities are comparable to the measured values across the full temperature range. For Per-PDA, the predicted values are larger than the measurements at low temperatures, but comparable at room temperature. MD simulations show that the thermal conductivity of Per-PDA is larger than that of TAPPy-PDA in both the in-plane and cross-plane directions. We hypothesize that reinforcing the cross-plane supramolecular interactions by increasing the size of the aromatic core at the node may lead to higher thermal conductivities. Although the measured *k*_⊥_ of Per-PDA is higher than that of TAPPy-PDA, the difference cannot be conclusively attributed to its chemical structure because differences in crystallite size and orientation may play a role. As higher-quality crystals or films of 2DPs become available, we expect that investigating these structure–property relationships will be critical. Overall, the MD simulations predict a larger thermal conductivity anisotropy than observed experimentally. For example, at room temperature, the in-plane thermal conductivity of Per-PDA is predicted to be seven times greater than its cross-plane thermal conductivity. This finding suggests the potential for greater measured anisotropy in more idealized experimental samples.

## Conclusions

Using FDTR and high-resolution calorimeters, we measured both in-plane and cross-plane thermal conductivities of two imine-linked 2DPs. The results from this study confirm the anisotropic thermal transport in this new materials class, which MD simulations have previously predicted. Variable-temperature measurements and MD simulations performed in this study support that the higher in-plane thermal conductivity is due to the stiff covalent bonds in the lateral 2DP plane, whereas the lower thermal conductivity in the cross-plane direction is a consequence of the weak, supramolecular interactions along this crystallographic axis. This investigation establishes 2DP as a unique class of synthetic organic nanomaterials with anisotropic thermal conductivities that may be engineerable with appropriate synthetic designs. A positive correlation between the experimental *k* and temperature is also observed, which suggests that the absolute magnitude of the thermal conductivity is limited by the polycrystalline nature of these 2DP thin films. While our measurements suggest that Per-PDA has a higher *k*_⊥_ than TAPPy-PDA, consistent with its more pronounced van der Waals surface interactions, it is challenging to definitively assign this structure–property relationship due to differences in the mesoscale features (e.g., grain boundaries, crystallite sizes, etc.) between these two materials. MD simulations further suggest that a perfectly oriented, bulk 2DP would exhibit higher in-plane thermal conductivity and greater anisotropy than observed in the experimental measurements. Going forward, higher quality 2DP samples that are synthetically engineered to provide high *k* may be useful in applications where lateral thermal dissipation is desired.

## Methods

### 2DP Thin-Film Interfacial Polymerization

A stock solution of the monomers were prepared by combining 1.56 mM of amine (TAPPy, 4.42 mg) or (Per, 4.81 mg) and 3.12 mM of PDA (2.09 mg) in 5 mL of THF:mesitylene (4:1, v/v). The stock solution was sonicated at room temperature until the monomers were fully dissolved. In a 20 mL scintillation vial, 0.1 mL of the stock solution was gently layered on top of an aqueous solution of Sc­(OTf)_3_ (5 mM, 2.0 mL). After a 30 min reaction time, water was added to the aqueous layer to raise the film closer to the opening of the scintillation vial. The film was gently transferred to a Petri dish containing MeOH. Then, the film was transferred onto a sapphire substrate or a copper TEM grid. After the film had dried, the films were washed gently with MeOH to remove excess starting material. See Section S1 for more details about synthesis and chemical characterization.

### FDTR

In FDTR, a continuous-wave (CW) laser at 488 nm (pump laser) is modulated by an electro-optic modulator (EOM) at frequencies ranging from 100 kHz to 5 MHz and focused onto the Au-coated sample surface to induce sinusoidal heating. The resulting temperature oscillates at the same frequency, with a phase lag determined by the sample’s thermal properties. A 532 nm CW probe laser detects this temperature modulation via the thermoreflectance change of the Au layer. The reflected signals from both pump and probe lasers are collected by a photodetector and fed to a lock-in amplifier to extract the phase lag between them. This frequency-dependent phase lag, sampled at 25 points between 100 kHz and 5 MHz, is fit to an analytical solution of the heat diffusion equation to extract thermal properties of the sample. See Section S2 for more details about the FDTR measurements and analysis.

### Suspended Calorimeter Platform

The temperature coefficient of resistance (TCR) of the platinum resistor was determined by measuring the resistance at temperatures ranging from 100 to 300 K. Next, the thermal frequency response of the suspended devices was measured to determine the time constant of the calorimeters and the corresponding frequency-dependent attenuation in the temperature rise. See Section S3.2 for additional details.

To determine the cross-conductance between the two membranes a known amount of power (1 μW – 2.5 μW) is dissipated in the heater membrane via Joule heating, by supplying an ac current of amplitude *I*_ac_ and a frequency (*f*) of 0.5 Hz. This frequency was chosen because it is much lower than the thermal roll-off frequency determined from the frequency response measurement, and thus a full thermal response is obtained. This power dissipation sets up temperature oscillations in the heater membrane at 2*f*. The amplitude of this temperature oscillation (Δ*T*_heater,2*f*_) is determined from the amplitude of voltage (Δ*V*_heater,3*f*_) at 3*f*, which was measured using a lock-in technique.^44^ The expression is given as

$$\Delta T_{\mathrm{heater},2f}=\frac{2\Delta V_{\mathrm{heater},3f}}{I_{\mathrm{ac}}R_{h}\alpha }$$ 2

where *R*_h_ is the electrical resistance of the heater and α is the TCR of the platinum resistor.

This temperature oscillation on the heater then couples into the sensor membrane through the film and also creates temperature oscillations on the sensor at 2*f*. A dc current (*I*_dc_) of 10 μA was driven across the sensor membrane and the amplitude of voltage oscillations (Δ*V*_sensor*,*2*f*_) at 2*f* was measured using lock-in detection. The amplitude of the temperature oscillations (Δ*T*_sensor,2*f*_) on the sensor was then determined using the following expression:

$$\Delta T_{\mathrm{sensor},2f}=\frac{\Delta V_{\mathrm{sensor},2f}}{I_{\mathrm{dc}}R_{s}\alpha }$$ 3

where *R*_s_ is the electrical resistance of the sensor.

The in-plane conductance (*G*_||_) across the membranes can be estimated by assuming a thermal model (see Figure S3.3) for the system. The expression for cross-conductance is given as follows:

$$G_{∥}=\frac{G_{\mathrm{th}}\times \Delta T_{\mathrm{sensor},2f}}{\left(\Delta T_{\mathrm{heater},2f}-\Delta T_{\mathrm{sensor},2f}\right)}$$ 4

where *G*_th_ is the beam conductance of the suspended membrane to the ambient and is assumed equal for both the membranes as both the devices are identical in all dimensions.

## Supplementary Material



## Notes

Synthesis (S1), characterization (S1), FDTR measurements (S2), suspended platform measurements (S3), and lattice dynamics calculations (S4) (PDF)
