# Supplementary material for: Anisotropic Thermal Conductivity in Imine-Linked Two-Dimensional Polymer Films Produced by Interfacial Polymerization
Source: ACS Nano. 2025 May 14;19(20):19009–17. doi: 10.1021/acsnano.4c17126 (PMC12120993; doi:10.1021/acsnano.4c17126)
Supplement: Supplementary file 1 [file nn4c17126_si_001.pdf]

Supporting Information

Anisotropic thermal conductivity in imine-linked 2D polymer films produced by interfacial polymerization

AUTHOR NAMES: Yuxing Liang[‡1], *Kiana A. Treaster*[‡2], *Ayan Majumder*[‡5], *Manoj Settipalli*[1], *Kanishka Panda*[5], *Shravan Godse*[1], *Rupam Roy*[2], *Ratul Mali*[5], *Zhongyong Wang*[5], *Yuxuan Luan*[5], *Peijie Hu*[4], *Keith Searles*[4], *David Charles McLeod*[6], *Kirt A. Page*[7,8], *Dayanni Bhagwandin*[7,8], *Edgar Meyhofer*[5], *Pramod Reddy*[5], *Alan J. H. McGaughey*[1], *Austin M. Evans**[2,3], *Jonathan A. Malen**[1]

[‡]*Authors contributed equally; *Corresponding Author*

[1] Department of Mechanical Engineering, Carnegie Mellon University, 5000 Forbes Ave, Pittsburgh, PA 15213, United States

[2] George and Josephine Butler Polymer Research Laboratory, Department of Chemistry, Center for Macromolecular Science and Engineering, University of Florida, Gainesville, Florida 32611, United States

[3] Department of Materials Science and Engineering, University of Florida, Gainesville, Florida 32611, United States.

[4] Center for Catalysis, Department of Chemistry, University of Florida, Gainesville, Florida, 32611, United States.

[5] Department of Mechanical Engineering, 2370 G.G. Brown Laboratory, University of Michigan, 2350 Hayward, Ann Arbor, MI 48109, United States

[6] DEVCOM Army Research Laboratory, Aberdeen Proving Ground, Maryland 21005, United States

[7] Materials and Manufacturing Directorate, Air Force Research Laboratory, WPAFB, Dayton, OH 45433, United States

[8] UES A BlueHalo Company, Dayton, OH 45432, United States

*jonmalen@andrew.cmu.edu

*austinevans@ufl.edu

1 Synthesis and chemical characterization

1.1 Materials and Instrumentation

Materials. 4,4',4'',4'''-(pyrene-1,3,6,8-tetrayl)tetrabenzaldehyde (TFPy), (1E,1'E,1''E,1'''E)-1,1',1'',1'''-(pyrene-1,3,6,8-tetrayltetrakis(benzene-4,1-diyl))tetrakis(N-(4-(tert-butyl)phenyl)methanimine) (TFPy-TBA),3,6,8-tetrakis(4-aminophenyl)pyrene (TAPPy), and 2,5,8,11-tetrakis(4-aminophenyl)perylene (Per) materials were prepared according to literature conditions. Reagents and solvents were purchased from commercial suppliers and used without further purification.

1.2 Instrumentation:

Nuclear Magnetic Resonance (NMR) Spectroscopy. ^{1}H NMR spectra of 2DP monomers were recorded on a Varian Inova 500 MHz spectrometer at ambient temperature. Deuterated DMSO-d_6 was used as an internal standard.

Powder X-ray Diffraction (PXRD). PXRD patterns were collected at the University of Florida Nanoscale Research Facility on a Panalytical X'pert Materials Research Diffractometer (MRD) (40 kV, 40 mA) equipped with Cu Kα1 radiation, λ = 1.54056 Å at room temperature. The patterns were recorded in the 2θ range of 2-30° and the scanning rate was 2 s per step with a step size of 0.1° for an overall exposure time of 10 mins.

Grazing-incidence wide angle X-ray scattering (GIWAXS). The grazing-incidence X-ray scattering measurements were carried out at the Functional Materials Beamline (FMB) of the Materials Solutions Network at the Cornell High Energy Synchrotron Source (MSN-C). An X-ray beam energy of 9.7 keV (λ=1.28 Å) was selected using the 111 reflection of a single-bounce, HPHT diamond monochromator. Harmonic rejection and vertical focusing are provided by a 1-meter long, bendable, rhodium-coated monochromatic mirror located approximately 7 meters upstream of the experimental hutch at an incident angle of 4 milliradians. Experiments were carried out in "bulk-beam" mode and the monochromatic mirror was used to focus the beam into a spot approximately 0.045 x 0.5 mm^2 at the sample position, with a total flux of approximately 10^{12} photons/second at 125 mA beam current. The samples were mounted on a 4-axis goniometer and aligned using a downstream ion chamber. Experiments were performed over a range of incident angles, both below and above the film critical angle. Scattering images were collected on a Pilatus 300K detector (Dectris, Baden, Switzerland) with a sample-to-detector distance of ca. 42.3 cm. Detector images were calibrated using silver behenate to convert the images to q-space. Python code was used to correct and analyze the scattering images and to produce intensity versus scattering vector, $Q_{||}$(Å^{-1}), plots.

Nitrogen Sorption Isotherms. Sorption isotherm measurements were collected on a Micromimetics ASAP 2020 Plus Accelerated Surface Area and Porosity analyzer. Approximately 30-50 mg of 2DP powder sample was transferred to a dried analysis tube and sealed with a Transeal cap. The sample was heated to 40 °C at a rate of 1°C min^{-1} and evacuated at 40°C for 20 min. Then, the sample was heated to 100°C at a rate of 1°C min^{-1} and evacuated at 100 °C until the

outgas rate was ≤0.3 μmHg min^{-1}. The tube was reweighed to determine the mass of the activated sample and subsequently transferred to the analysis port to begin analysis. N_2 was used for all adsorption measurements. N_2 isotherms were generated by incremental exposure to nitrogen up to 760 mmHg (1 atm) in a liquid nitrogen (77 K) bath. Brunauer-Emmett- Teller (BET) surface areas were calculated from the linear region of the N_2 isotherm.

Atomic Force Microscopy (AFM). AFM was conducted on an AFM Workshop TT-2 AFM using a silicon tip under the non-contact mode. Images were collected in air.

1.3 Monomer Synthesis

All reactions were performed using oven-dried glassware under inert conditions.

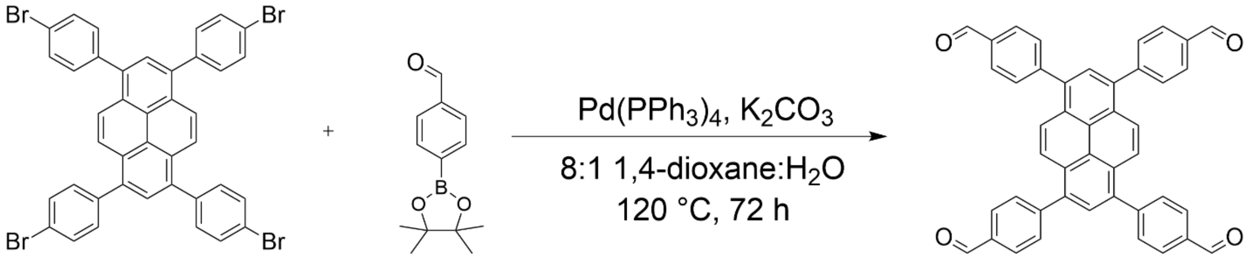

Model compound monomer (TFPy). This synthesis was adapted from a previous report. In a 50 ml Schlenk round bottom flask, 1,3,6,8-tetrabromopyrene (750 mg, 1.448 mmol, 1.0 equiv.), 4-formyl phenyl boronic acid pinacol ester (1.68 g, 7.238 mmol, 5.0 equiv.), $Pd(PPh_3)_4$ (165 mg, 0.143 mmol, 0.098 equiv.) and K_2CO_3 (1.15 g, 8.320 mmol, 5.7 equiv.) were taken and properly charged for 30 min following vacuum and N_2 flow method. To this mixture, 1,4-dioxane (16 ml) and H_2O (2 ml) were added and kept at 120 °C (reflux condition) for 72 h. After completion of the reaction, H_2O was added resulting in a precipitate formation. The precipitate was filtered and subsequently washed with H_2O and MeOH. The solid residue was dried in air and purified by recrystallization in 1,4-dioxane solvent. The pure compound was obtained as a yellow powder (83% yield). ^{1}H NMR of this compound was consistent with previous reports.

^{1}H NMR (500 MHz, $CDCl_3$): 10.16 (s, 4 H), 8.18 (s, 4 H), 8.09 (d, J = 7.8 Hz, 8 H), 8.04 (s, 2 H), 7.86 (d, J = 8.0 Hz, 8 H), 3.70 (s, 12 H, dioxane).

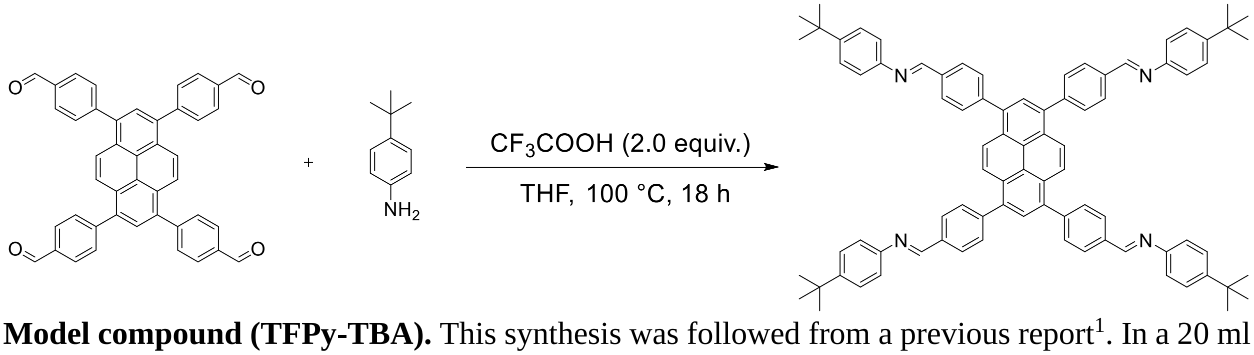

Model compound (TFPy-TBA). This synthesis was followed from a previous report[1]. In a 20 ml vial, TFPy (50 mg, 0.081 mmol, 1.0 equiv.) and TBA (72 mg, 77 mL, 0.483 mmol, 6.0 equiv.) were dissolved in 6 ml THF and mixed by sonication. To this mixture, TFA (18.47 mg, 13 mL, 0.162 mmol, 2.0 equiv.) was added and the reaction mixture was stirred at 100 °C for 18 h. After

completion, the reaction mixture was neutralized with aqueous K_2CO_3 solution and followed by washing the organic layer with water (2 times) and brine solution (2 times). The solution was dried over $MgSO_4$ and concentrated under reduced pressure. The residue was purified by column using DCM as eluent.

^{1}H NMR (500 MHz, $CDCl_3$): 8.62 (s, 4 H), 8.23 (s, 2 H), 8.15 – 8.04 (m, 12 H), 7.81 (d, J = 8.3 Hz, 8 H), 7.45 (s, 8 H), 7.25 (d, J = 2.2 Hz, 8 H), 1.37 (d, J = 4.2 Hz, 36 H).

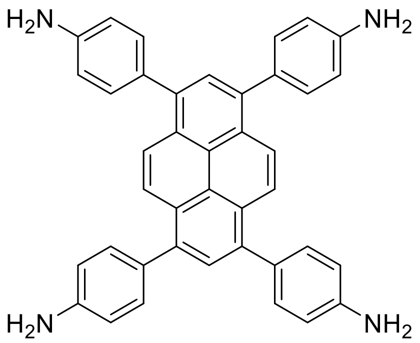

1,3,6,8-tetrakis(4-aminophenyl)pyrene (TAPPy). This synthesis was followed from a previous report. To a flame-dried flask, 1,3,6,8-tetrabromopyrene (1482 mg, 2.86 mmol, 1.0 eq.), 4-aminophenylboronic acid pinacol ester (3010 mg, 13.7 mmol, 4.8 eq.), K_2CO_3 (2175 mg, 15.7 mmol, 5.5 eq.) and $Pd(PPh_3)_4$ (330 mg, 0.29 mmol, 10 mol%) was added to 32 mL 1,4-dioxane and 8 mL H_2O, then heated to reflux (115 °C) for 3 days. After cooling to room temperature, H_2O was added, and the precipitate was collected by filtration and washed with H_2O and MeOH sufficiently. The resulting precipitate was recrystallized in 1,4-dioxane solvent, followed by drying under high vacuum yielding in a yellow-green powder (71% yield). ^{1}H NMR of this compound was consistent with previous reports.

^{1}H NMR (500 MHz, DMSO-d_6): 8.12 (s, 4 H), 7.81 (s, 2 H), 7.33 (d, J = 8.4 Hz, 8 H), 6.77 (d, J = 8.5 Hz, 8 H), 5.28 (s, 8 H), 3.57 (s, 12 H, dioxane).

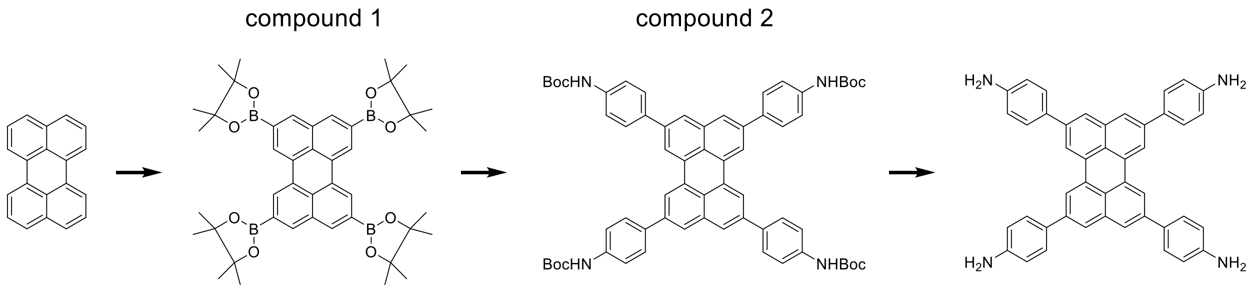

S1.1. Synthesis of 2,5,8,11-tetrakis(4-aminophenyl)perylene (Per) monomer[2]

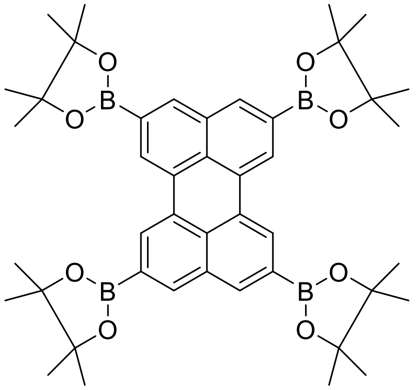

2,5,8,11-tetrakis(4,4,5,5-tetramethyl-1,3,2-dioxaborolan-2-yl)perylene (compound 1[2]). This synthesis was adapted from a previous report. To a flame-dried flask, perylene (2019 mg, 8.00 mmol, 1.0 eq.), $[Ir(OMe)(1,5\text{-cod})]_2$ (265 mg, 0.40 mmol, 5 mol%), 4,4′ - di-*tert*-butyl-2,2′ - bipyridyl (215 mg, 0.80 mmol, 10 mol%), and bis(pinacolato)diboron (9751 mg, 38.4 mmol, 4.8 eq.) was added to 64 mL anhydrous cyclohexane and stirred under nitrogen at 80 °C for 40 h. After cooling to room temperature, the yellow precipitate was collected by filtration, washed with cyclohexane and acetone subsequently, yielding compound **1** as a bright yellow powder (91% yield). The material was used directly in the next step without further purification. ^{1}H NMR of this compound was consistent with previous reports.

^{1}H NMR (500 MHz, $CDCl_3$): 8.62 (s, 4 H), 8.25 (s, 4 H), 1.43 (s, 48 H).

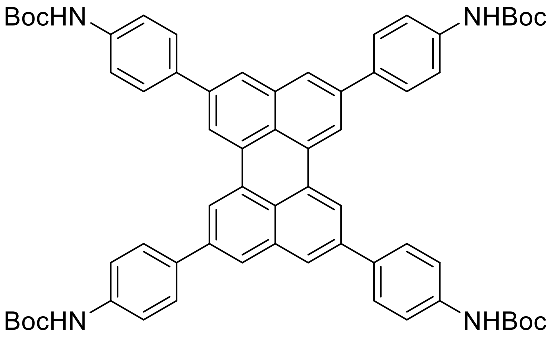

2,5,8,11-tetrakis(4-Boc-aminophenyl)perylene (compound 2[2]). This synthesis was adapted from a previous report. To a flame-dried flask, compound **1** (1512 mg, 2.00 mmol, 1.0 eq.), N-Boc-4-bromoaniline (8708 mg, 32.0 mmol, 16 eq.), K_2CO_3 (2211 mg, 16.0 mmol, 8.0 eq.), $Pd_2(dba)_3 \cdot CHCl_3$ (414 mg, 0.40 mmol, 20 mol%), and Sphos (328 mg, 0.80 mmol, 40 mol%) was added to 64 mL *o*-xylene and 16 mL H_2O was stirred under nitrogen at 100 °C for 40 h. After cooling to room temperature, 20 mL MeOH was added, and the precipitate was collected by filtration and washed with H_2O and MeOH sufficiently. The green solid was dissolved in a 6:4 DCM/EtOAc mixture and passed through a silica plug. The solution was concentrated under reduced pressure, to precipitate the product. The precipitate was collected by filtration and washed successively with 10 mL MeOH and 10 mL DCM. For further purification, the product was dispersed in 600 mL EtOAc and sonicated for 30 min. The suspension was concentrated, and the solids were collected by filtration. Then, the solids were washed sufficiently with 10 mL EtOAc

and 10 mL DCM, and dried under high vacuum to yield compound **2** as a greenish-yellow powder (59% yield).^{1}H NMR of this compound was consistent with previous reports.

^{1}H NMR (500 MHz, DMSO-d_6): 9.52 (s, 4 H), 8.73 (s, 4 H), 8.14 (s, 4 H), 7.92 (d, J = 8.6 Hz, 8 H), 7.66 (d, J = 8.3 Hz, 8 H), 1.52 (s, 36 H)

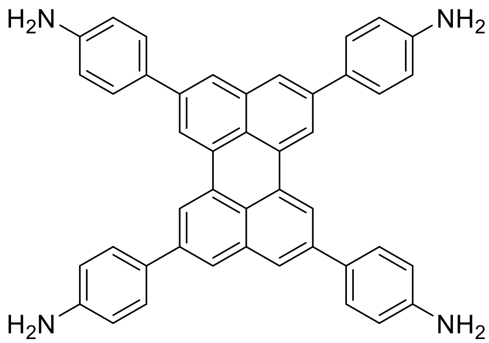

2,5,8,11-tetrakis(4-aminophenyl)perylene (Per[2]). This synthesis was adapted from a previous report. A suspension of compound **2** (829 mg, 0.82 mmol, 1.0 eq.) in 30 mL anhydrous DCM was made, followed by the addition of 10 mL TFA. The dark yellow solution was stirred for 2 h at room temperature. The reaction mixture was neutralized by slowly adding a solution of saturated $NaHCO_3$ solution in purged H_2O, resulting in a dark orange solid. The solids were collected by filtration, washed with 500 mL purged H_2O and dried under high vacuum to yield the compound as a dark solid (92% yield). ^{1}H NMR of this compound was consistent with previous reports.

^{1}H NMR (500 MHz, DMSO-d_6): 8.54 (s, 4 H), 7.95 (s, 4 H), 7.69 (d, J = 8.4 Hz, 8 H), 6.75 (d, J = 8.6 Hz, 8 H), 5.32 (s, 8 H).

1.4 2DP Powder Synthesis

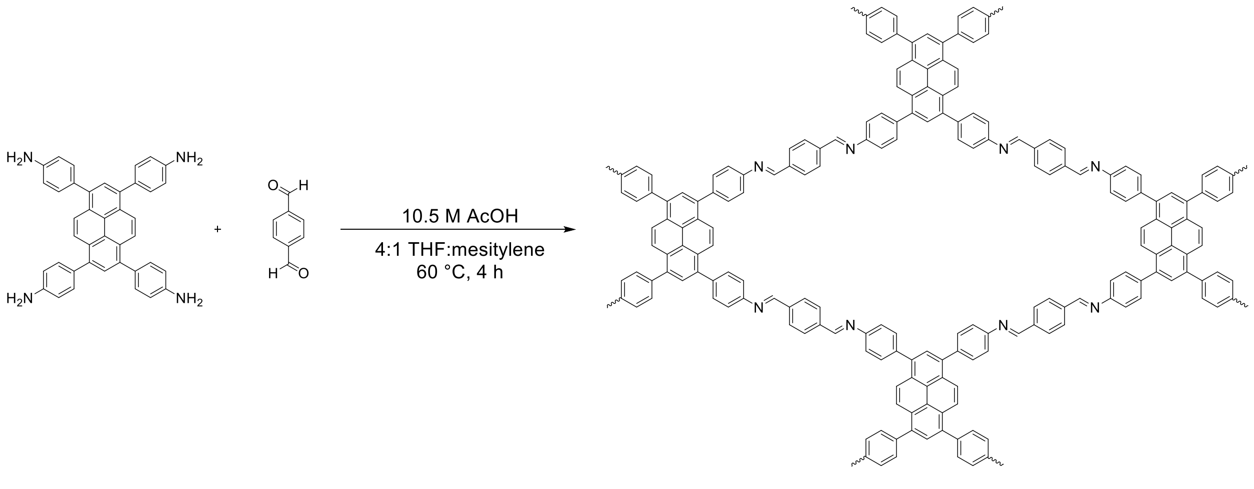

S1.2. Synthesis of TAPPy-PDA 2DP powder

TAPPy-PDA[3]. To synthesize TAPPy-PDA 2DP powder, 56.67 mg of TAPPy (0.1 mmol) and 26.83 mg of terepthaldehyde (PDA, 0.2 mmol) was added to a 4 mL vial. 1 mL of 4:1

THF:mesitylene was added to the vial and the mixture was sonicated for 1-2 minutes. The vial was preheated to 60 °C using an aluminum heating block. To the preheated solution, 0.5 mL of 10.5 M acetic acid was added. The vial was capped securely, then gently swirled to mix the acid into the reaction mixture, then the reaction mixture was allowed to stand for 4 hours at 60 °C. After the specified reaction time, the 2DP reaction mixture was diluted with methanol, gently swirled, and the supernatant was decanted 2-3 times. The methanol-soaked 2DP solids were transferred to a tea bag then washed with more methanol. While wet, the 2DP was transferred into a 20 mL scintillation vial and dichloromethane (DCM), (ca. 10 mL) was added to it. After 1 h, DCM was decanted and replaced with fresh DCM. This process was repeated twice. Subsequently, the DCM was decanted and replaced with n-hexane. At the interval of 1 h, the n-hexane was replaced with fresh solvent three times. Then the solid was placed in a clean vial and heated to 150 °C under nitrogen flow for 1 hour to remove n-hexane. This results in a fluffy yellow solid in 90% yield.

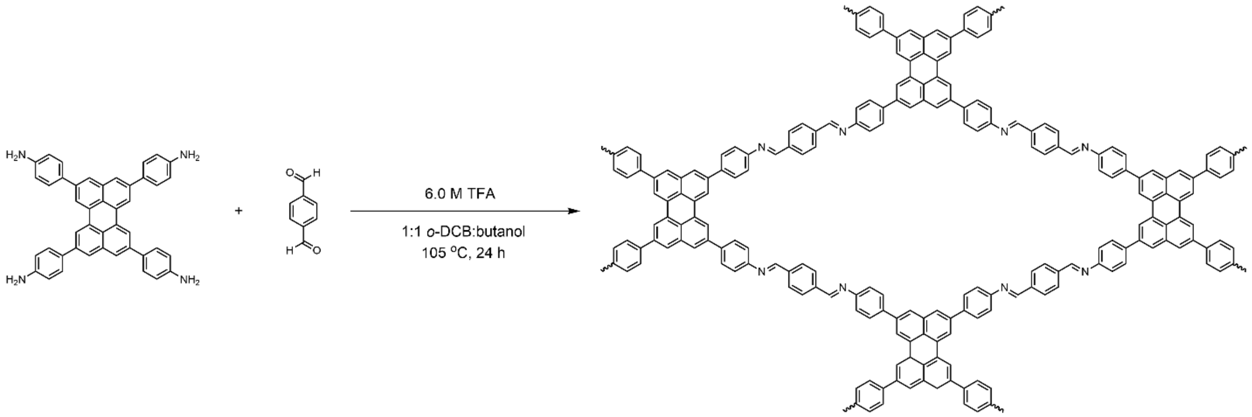

S1.3. Synthesis of Per-PDA 2DP powder

Per-PDA. To synthesize Per-PDA 2DP powder, 61.68 mg Per (0.1 mmol) and 26.83 mg of PDA (0.2 mmol) was added to a 20 mL scintillation vial. 3 mL of 1:1 *o*-dichlorobenzene:butanol was added to the vial and the mixture was sonicated for 30 minutes. Then, 0.2 mL of trifluoroacetic acid (TFA) was added to the mixture and the reaction mixture was heated to 105 °C overnight using an aluminum heating block. After the specified reaction time, the 2DP powder was removed from the heating block and allowed to cool to room temperature. Once cooled, the 2DP powder was filtered and washed thoroughly with dimethyl formamide (DMF), (ca. 100 mL). At this stage, do not allow the 2DP to become dry on the filter paper. After washing, the damp 2DP powder was transferred to a tea bag and washed with DCM using a Soxhlet extractor for 12 hours. The 2DP solid was immersed in n-hexane for three times with 1 hr interval in Per-PDA synthesis. Then the solid was placed in a clean vial and heated to 150 °C under nitrogen flow for 1 hour to remove n-hexane. This results in a deep brown solid in 87% yield.

1.5 2DP Thin-film Interfacial Polymerization

General Procedure[4]**.** A stock solution of the monomers were prepared by combining 1.56 mM of monomer (TAPPy, 4.42 mg) or (Per, 4.81 mg) and 3.12 mM of PDA (2.09 mg) in 5 mL of THF:mesitylene (4:1, v/v). The stock solution was sonicated at room temperature until the monomers were fully dissolved. In a 20 mL scintillation vial, 0.1 mL of the stock solution was gently layered on top of an aqueous solution of $Sc(OTf)_3$ (5 mM, 2.5 mL). After a 30-minute

reaction time, water was added to the aqueous layer to raise the film closer to the opening of the scintillation vial. The film was gently transferred to a petri dish containing MeOH. Then, the film was transferred onto a sapphire substrate or a copper TEM grid. After the film had dried, the films were washed gently with MeOH to remove excess starting material.

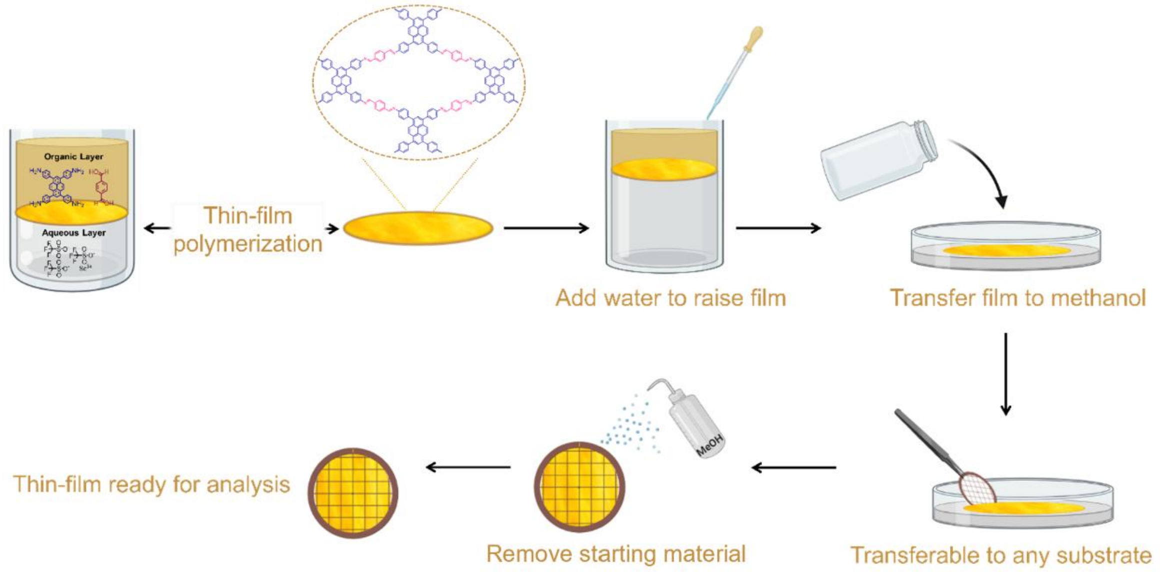

S1.4. Interfacial polymerization scheme of TAPPy-PDA 2DP

1.6 NMR Spectra

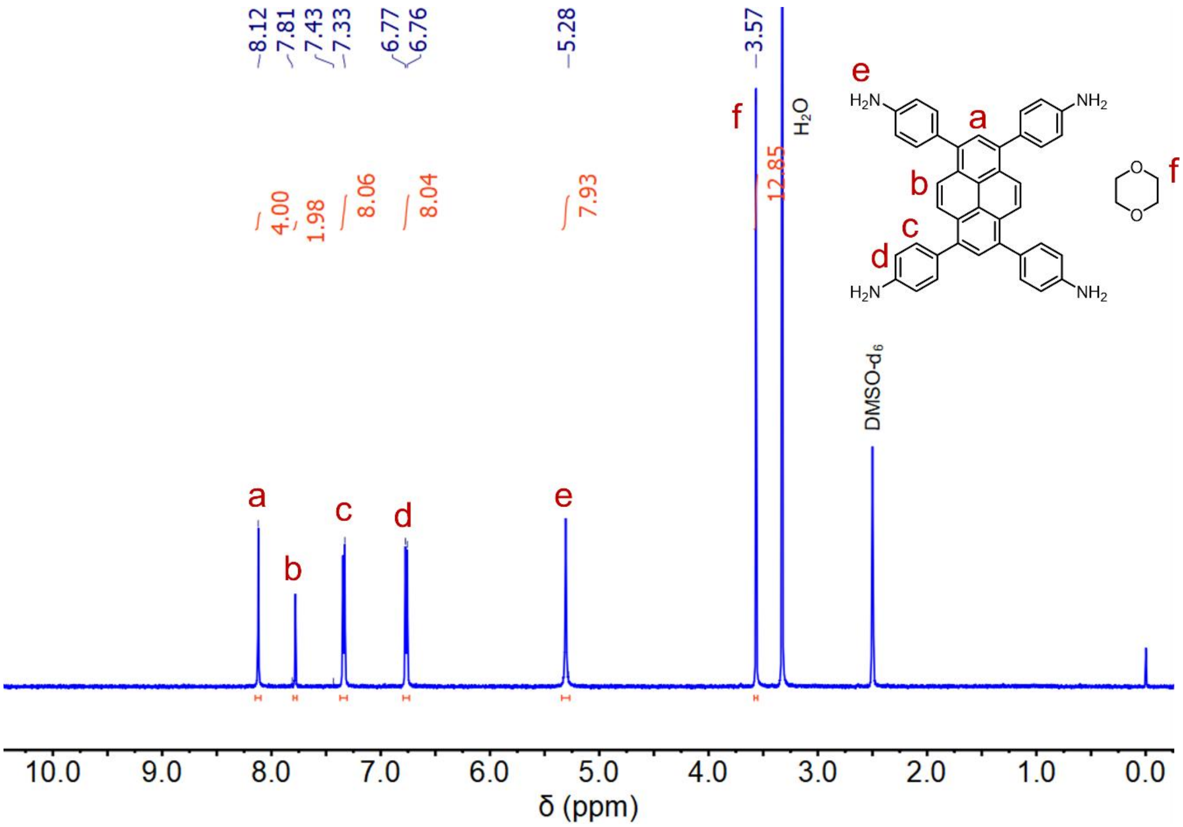

S1.5.1. ^{1}H NMR of TAPPy monomer

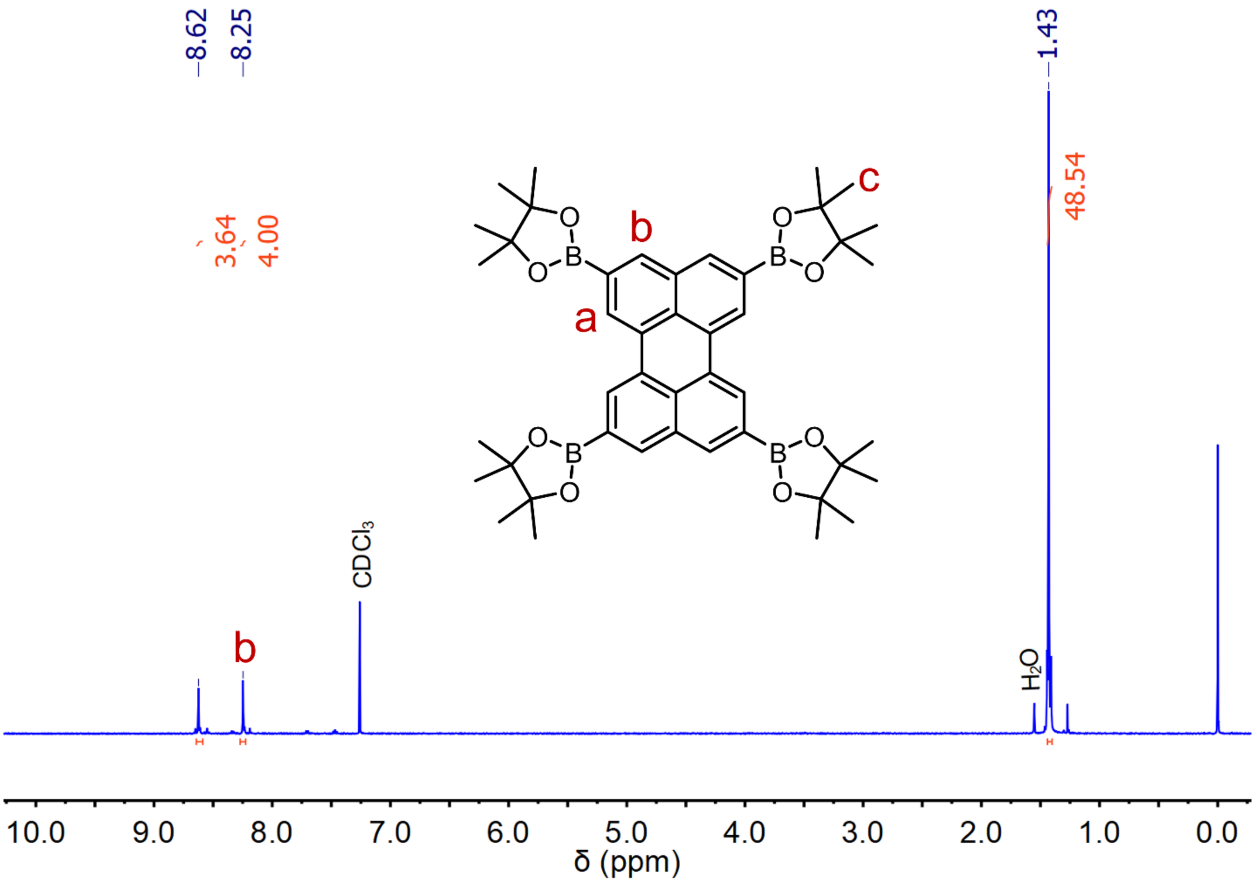

S1.5.2. ^{1}H NMR spectra of compound **1**

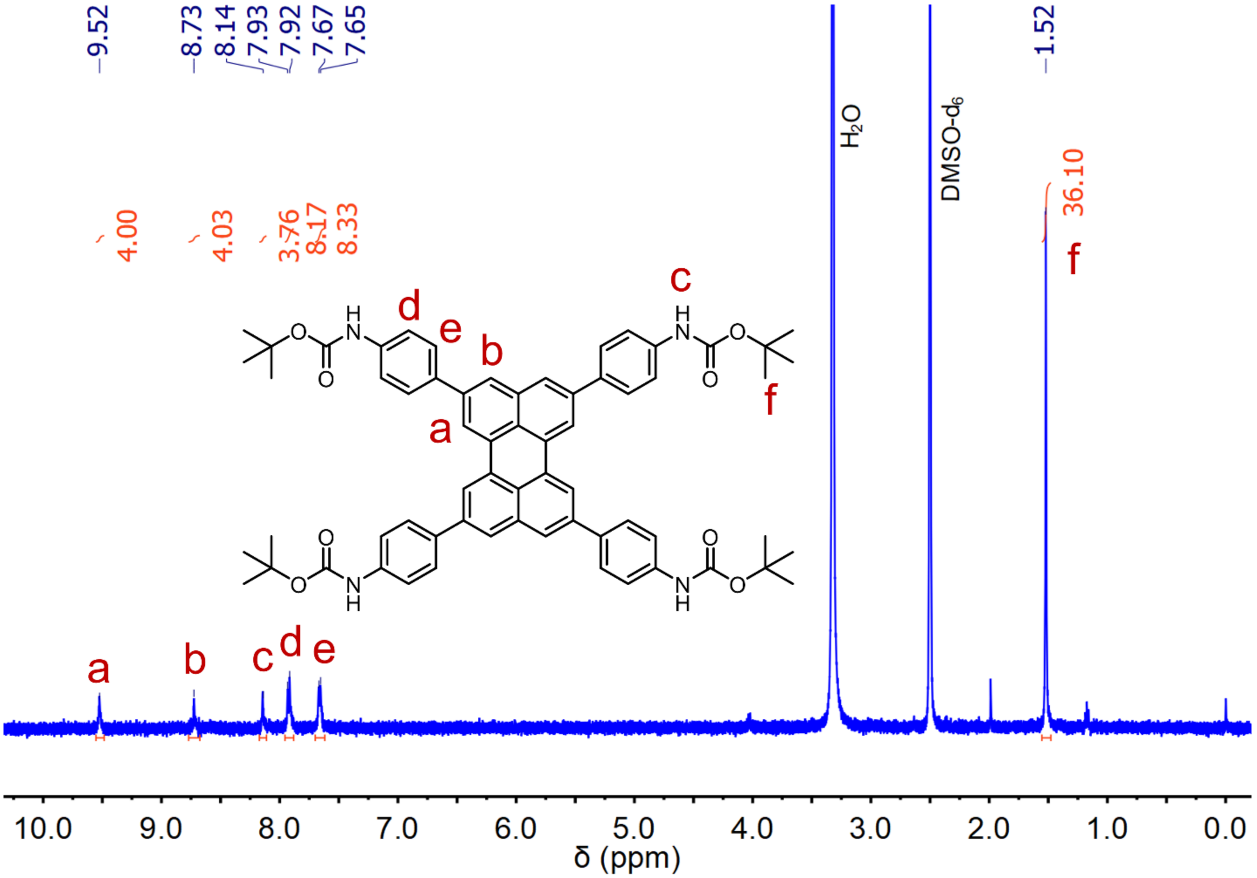

S1.5.3. ^{1}H NMR spectra of compound **2**

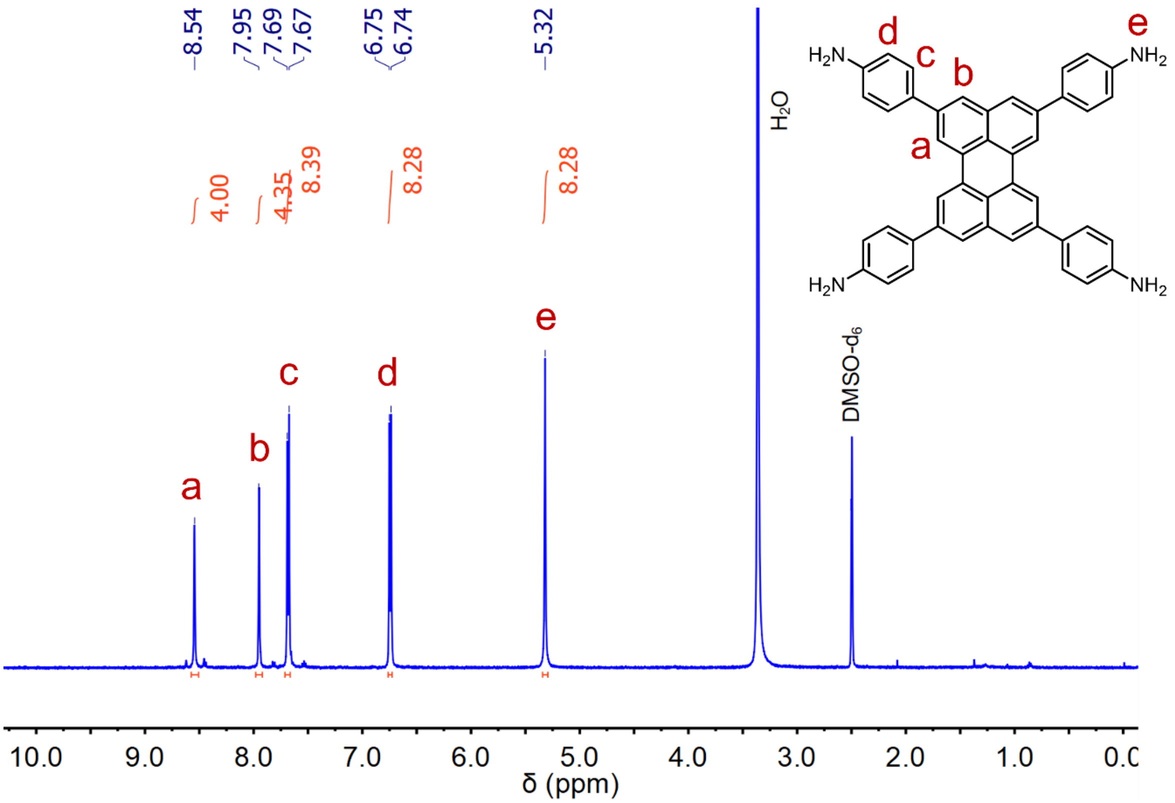

S1.5.4. ^{1}H NMR spectra of Per monomer

1.7 XRD

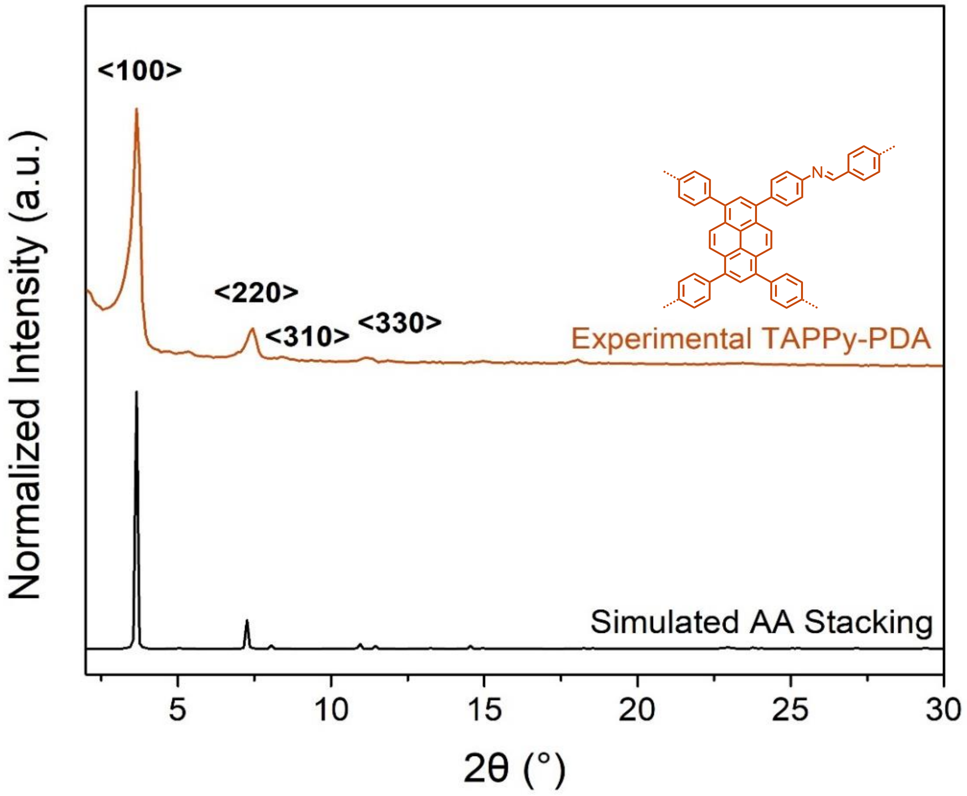

S1.6 PXRD of TAPPy-PDA 2DP. Simulated diffraction patterns

1.8 Nitrogen Sorption Isotherms

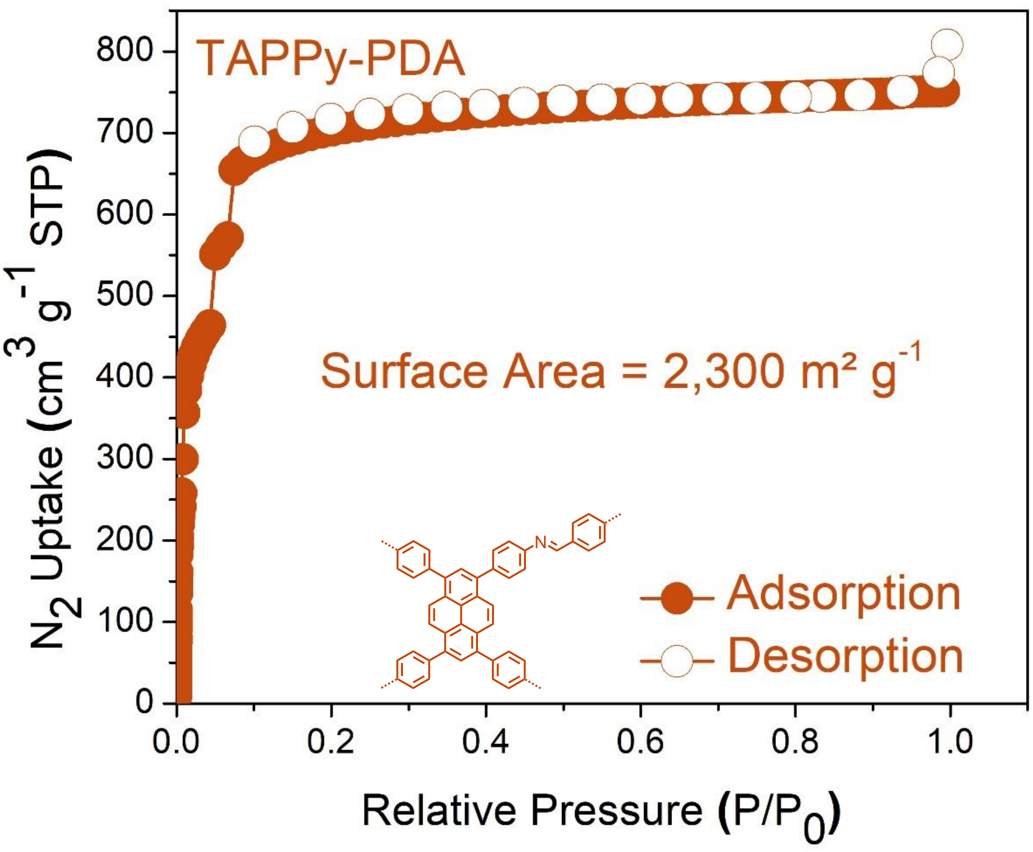

S1.7 N_2 sorption isotherm of TAPPy-PDA powder

1.9 AFM

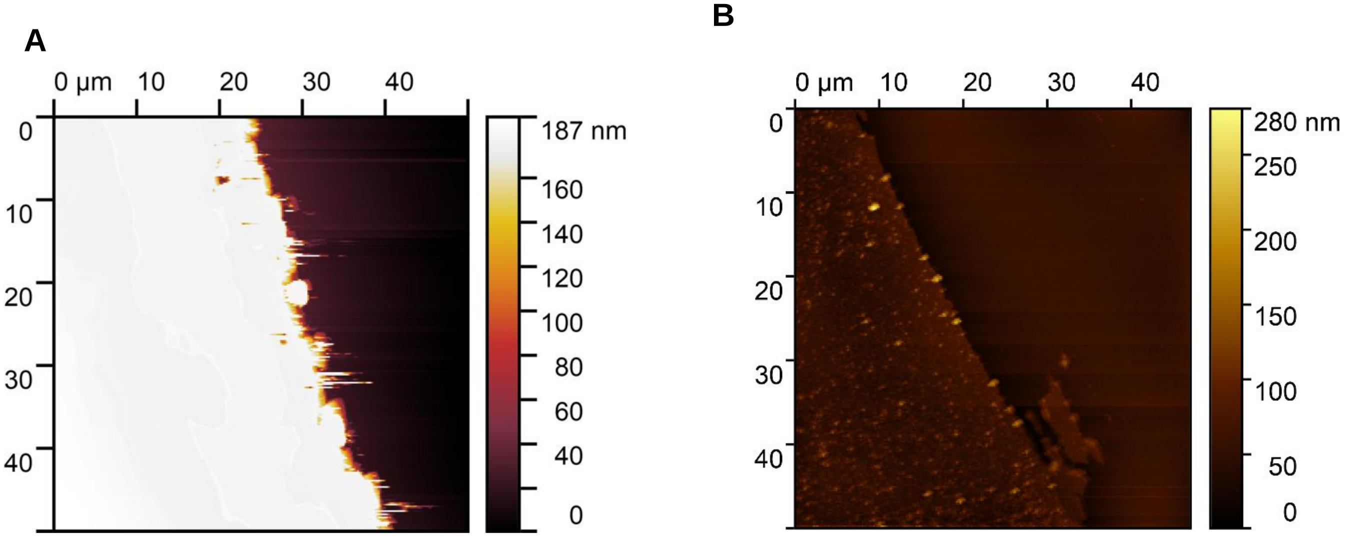

S1.8. AFM images **A.** TAPPy-PDA thin-film (thickness 156 nm, root mean squared roughness 10-20 nm) **B.** Per-PDA thin-film (thickness 36 nm, root mean squared roughness 5-15 nm)

2 Frequency Domain Thermoreflectance

2.1 Experimental setup (FDTR)

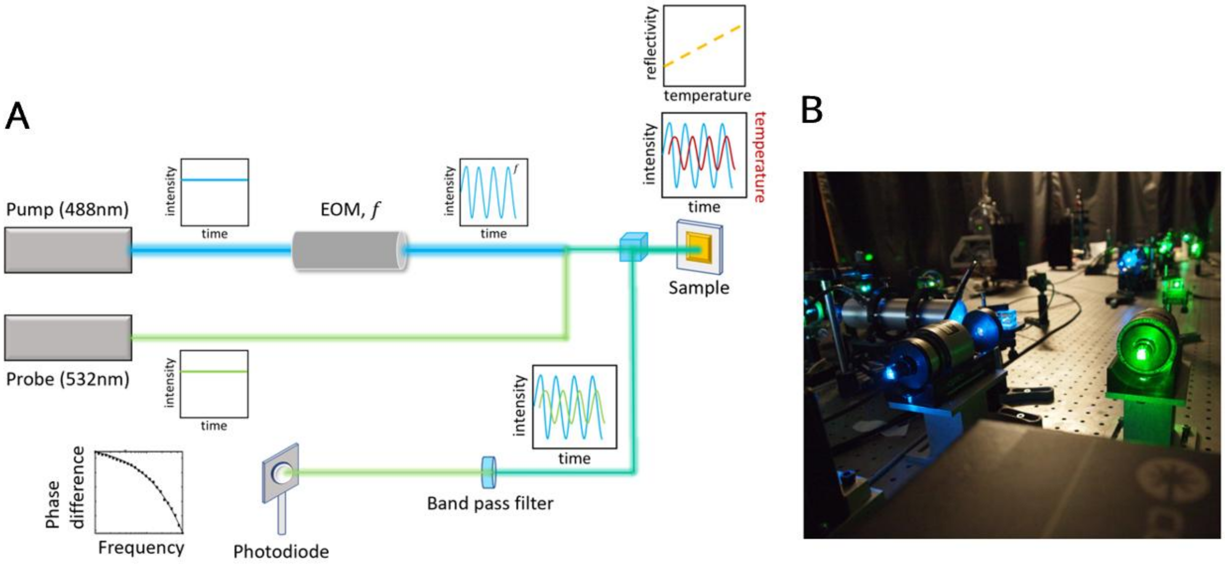

Figure S2.1 a. FDTR schematics; b. FDTR setup in the lab.

Frequency domain thermal reflectance (FDTR) is a laser based non-contact technique that does not require complex sample preparation and is highly sensitive to thermal conductivity in ultrathin samples that conventional techniques typically are not[5,6]. The samples are coated with 80 nm of Au in order to create the desired optical properties for FDTR. In FDTR, a continuous wave laser with a wavelength of 488 nm (called the pump laser) will be modulated by the electro-optic modulator (EOM) at frequency from 100 kHz to 5 MHz. This laser is focused onto the Au coated sample to sinusoidally heat the surface. In response, the temperature of the sample will periodically oscillate at the same frequency as the pump laser but with a phase lag related to the sample's thermal transport properties. The periodic oscillation of the sample temperature will be sensed by a continuous wave laser with a wavelength of 532 nm (the probe laser). The probe laser senses temperature due to the thermoreflectance of the Au coating. The pump and probe lasers will be collected by a photodetector and fed to a lock-in amplifier, which detects the phase lag of the probe to the pump. The phase lag is a function of frequency and thermal properties of the samples. The phase lag data detected at different frequencies (sampled from 100 kHz to 5 MHz) will be fit by an analytical model to the heat diffusion equation to determine the target parameters. [7]

2.2 Analytical Model (FDTR) and sensitivity check

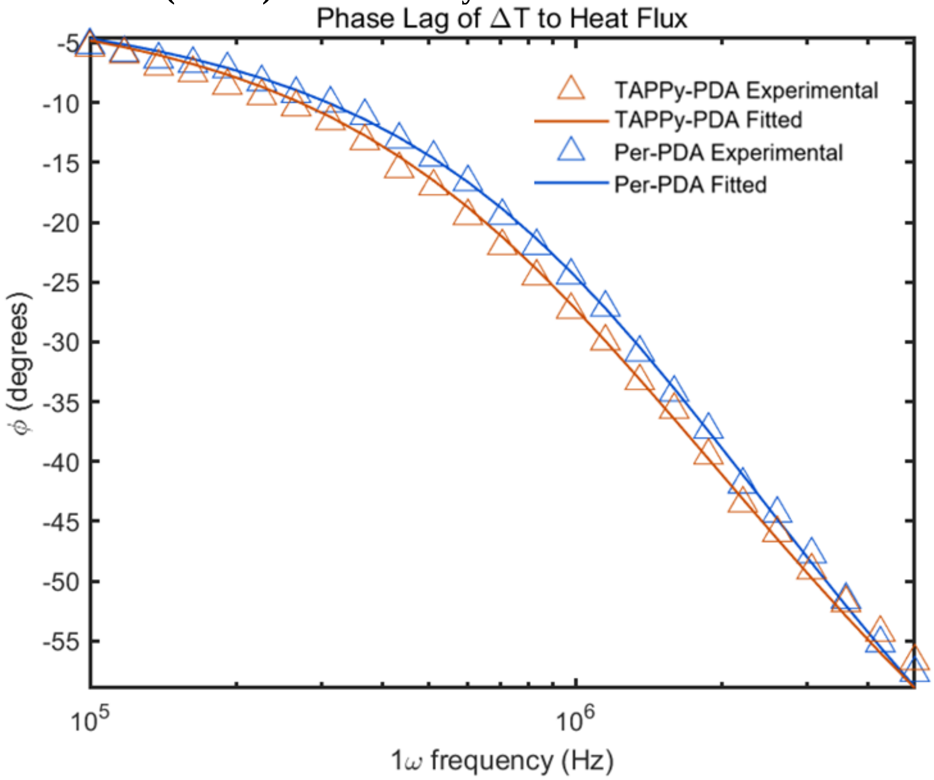

Figure S2.2 FDTR phase difference vs. frequency. The discrete data acquired from FDTR (red) are fit to the MSE minimizing solution of the heat diffusion equation to determine the sample's thermal conductivity. The solid line is the fitted phase difference. Red denotes the dataset and fitting for the TAPPy-PDA thin film, while blue corresponds to the Per-PDA thin film.

By defining the sensitivity as:

$$S_{\emptyset} = \frac{\beta}{\phi}\frac{\partial \phi}{\partial \beta} \tag{2.1}$$

$S_{\emptyset}$ is the normalized derivative of the phase lag, ϕ, with respect to a change in one of the key parameters $\beta = k_{\perp}, k_{\parallel},\ C,\ L, r_{spot}$ (cross-plane thermal conductivity, in-plane thermal conductivity, heat capacity, thickness of the polymer thin film).

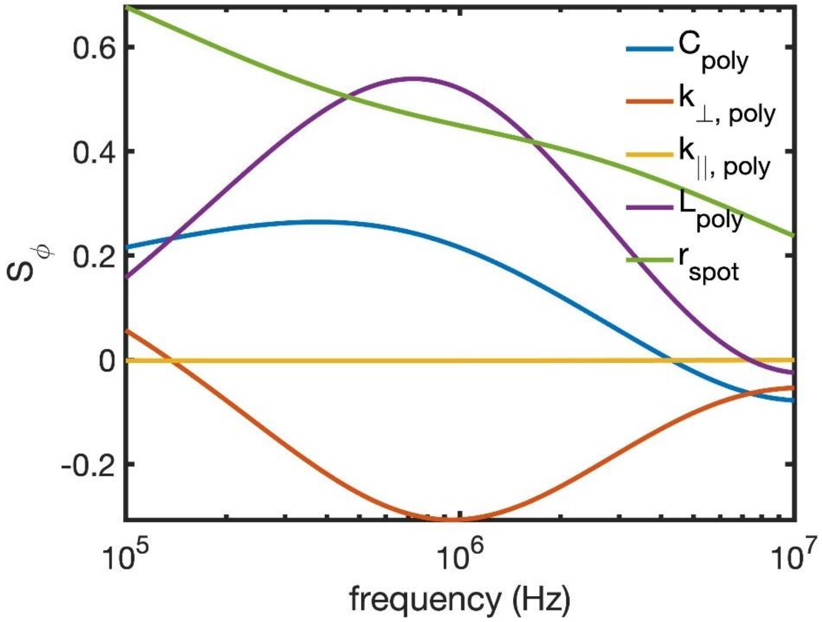

Figure S2.3 Sensitivity check of the polymer thin film.

The sensitivity for a sample comprised of 80 nm of Au on a 200 nm polymer thin film on an Al_2O_3 wafer is shown in Figure S2.3. The model exhibits minimal sensitivity to the in-plane thermal conductivity of polymer thin film. This is because although the 2DPs are anisotropic, the thermal resistance for heat to flow cross-plane into the high thermal conductivity substrate is much lower than for heat to spread in-plane in the 2DP. Hence, FDTR is a measure of $k_{\perp}$. Also, the model exhibits substantial sensitivity to the laser spot size, surpassing the impact of other parameters. This sensitivity is not necessarily concerning, however, since the spot size is well-understood and is not expected to vary significantly to introduce error in the fit of thermal conductivity. It is noteworthy that parameters unknown at the time of measurement, specifically the sample heat capacity and the sample thickness should be predetermined as accurately as possible to minimize their impact on the quality of fit.

Measurements of this nature are routinely made using thermoreflectance-based methods like FDTR and time-domain thermoreflectance (TDTR).[8,9] In order to have sensitivity to the unknown film it should have a thermal resistance greater than or comparable to the other thermal resistances in the system.

- Au Film: The Per-PDA thin film presented in maintext has a thickness of 126 nm while that of TAPPy-PDA is 164 nm. Since Au thermal conductivity is ~150 W/m-K, its thermal resistance (*L/k*) is more than 100 times smaller than the 2DP thin films, which have $k_{\perp}$~0.2 W/m-K.
- Al_2O_3 Substrate: Although the substrate is very thick, the periodic nature of the FDTR heating creates a finite thermal penetration depth $L_p = \sqrt{k/\pi f C}$ where k and C are the thermal conductivity and volumetric heat capacity of Al_2O_3, and f is the modulation frequency.[8,9] The thermal resistance of the substrate can be estimated as L_p/k. For f = 1

MHz, this thermal resistance is approximately 5×10^{-8} m^2-K/W. The thermal resistance of the 2DP film is approximately 5×10^{-7} m^2-K/W, which is 10 times larger.

Hence, in these measurements 2DP thin films have large thermal resistance compared to other resistances in the system. FDTR is thereby sensitive to the cross-plane thermal conductivity of the 2DP thin films.

2.3 FDTR fitting parameters

The nominal values and uncertainties for the samples measured by FDTR are shown in the table.

	2DP thickness (nm)*	2DP Heat Capacity (MJ/m^3-K)	Au thickness (nm)**	Au k (W/m-K)***	Sapphire k (W/m-K)	r_{spot} (μm)
TAPPy-PDA	164±31	0.50	78±2	151±9	38	3.4±0.2
Per-PDA	124±2	0.52	78±2	113±8	38	3.4±0.2

*Measured by profilometer (example below)

**Measured by X-ray reflectivity

***Measured electrical conductivity by 4-pt probe directly on sample and converted to thermal conductivity using the Wiedemann-Franz Law. The electrical resistivity of Au deposited on 2DPs was not measured directly at low temperatures. Temperature-dependent data for Au thin films of similar thickness and deposition conditions,[10] was scaled based on our room temperature measurements. This approach was validated through low-temperature FDTR measurements of Au on SiO_2 reference samples. Additionally, a 10% variation in Au thermal conductivity results in only a 5% change in the 2DP thermal conductivity, indicating that our FDTR measurements are not highly sensitive to the Au thermal conductivity. This uncertainty is included in the reported error bars on the temperature dependent data.

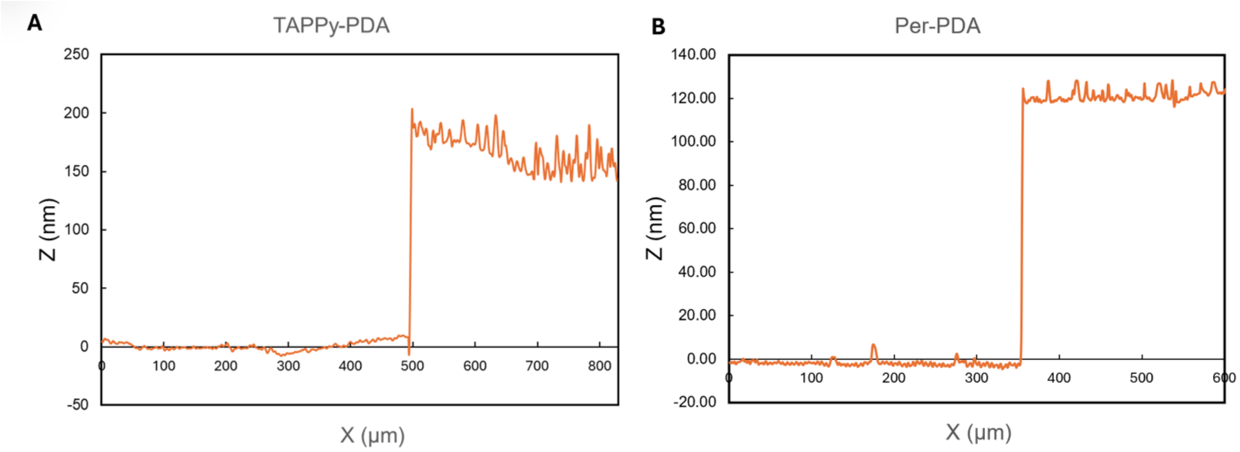

Figure S2.4. Profilometer scan on **A**. TAPPy-PDA thin film; **B**. Per-PDA thin film.

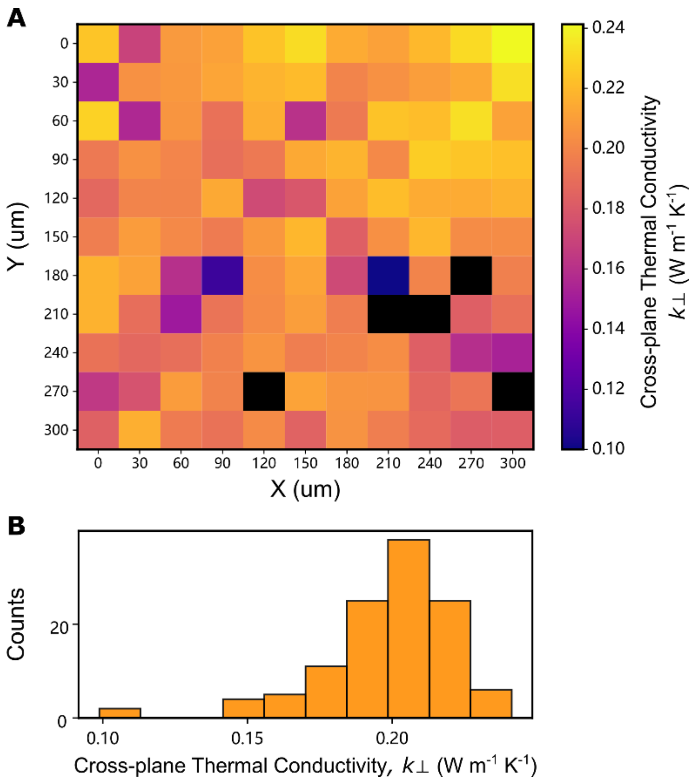

Figure S2.5. A. Heatmap of FDTR scanning over a 300 µm × 300 µm area of TAPPy-PDA thin film; The black squares are the filtered measurements with unsatisfactory fits (See SI Section 2.2). **B.** Histogram summarizing the cross-plane thermal conductivities from 116 individual measurements on a single TAPPy-PDA thin film.

2.4 Negligible impact of thermal conductance

Figure S2.6 subplots A and B show the FDTR scan for a Per-PDA thin film with a thickness of 124 nm, which yields a thermal conductivity of $\mathbf{0.24{\pm}^{0.03}_{0.03}}$ **W/m-K**, while subplots C and D show the FDTR scan for thickness of 156 nm which yields a thermal conductivity of $\mathbf{0.22{\pm}^{0.03}_{0.04}}$ **W/m-K**. FDTR measurements on Per-PDA thin films with different thicknesses yield consistent results, indicating that the extracted thermal conductivity is independent of thickness as shown by the heatmaps below. Consider a film with thickness L and an effective thermal conductivity k_{eff} that includes the effects of TBC. Its thermal resistance L/k_{eff} can be equated to the thermal resistance of a film of thickness L with intrinsic thermal conductivity $k_{\mathrm{intrinsic}}$ plus the separate resistance due to TBC: $\frac{L}{k_{eff}} = \frac{L}{k_{intrinsic}} + \frac{1}{TBC}$. If the resistance due to TBC were comparable to the intrinsic film resistance, we should see k_{eff} increase with L, which we do not observe. Hence, TBC has not been included in our analysis of the data.

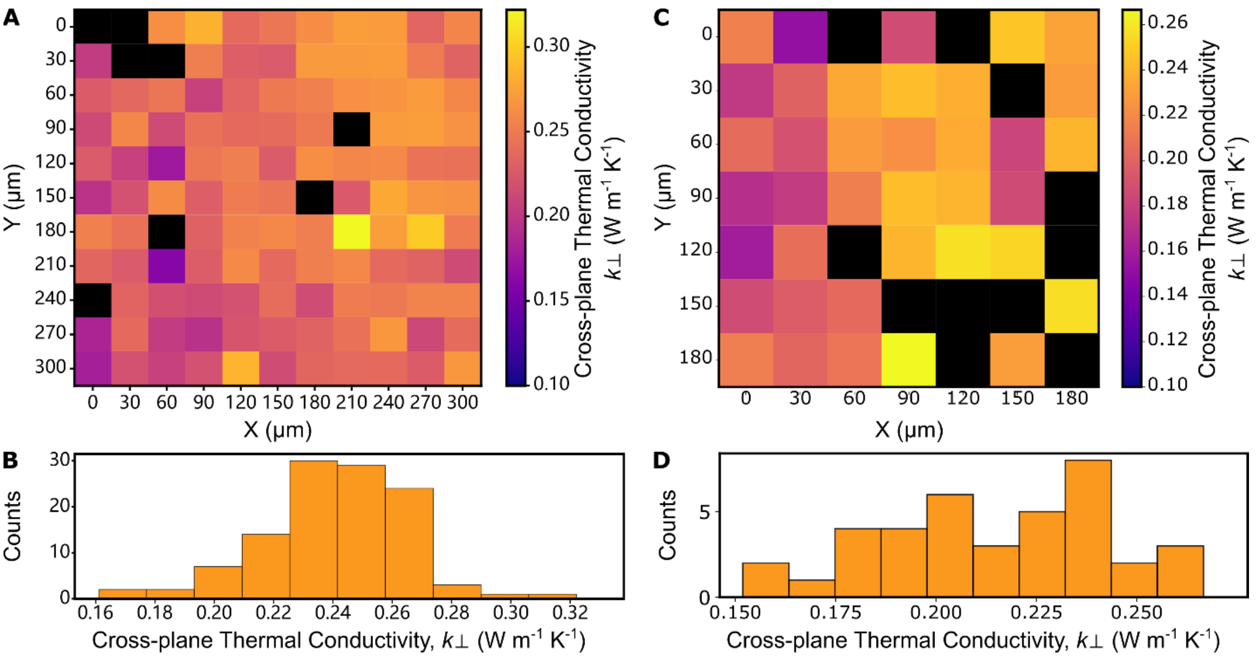

Figure S2.6. A, C. Heatmap of FDTR spatial scan on Per-PDA thin films with thickness of 124 nm (**A**) and thickness of 156 nm (**C**); The black squares are the filtered measurements with unsatisfactory fits (See SI Section 2.2). **B, D.** Histogram summarizing the cross-plane thermal conductivities on Per-PDA thin films with thickness of 124 nm (**B**) and thickness of 156 nm(**D**).

3 Suspended Platform

3.1 In-plane thermal conductivity measurements of TAPPy-PDA and Per-PDA samples.

High-resolution calorimetry has been implemented in the past for a wide range of studies in nanoscale heat transfer[11–16]. We designed and fabricated high-resolution calorimeters[17,18] to perform the in-plane thermal conductivity measurements. The devices, made of silicon nitride (SiN), consist of two suspended, coplanar membranes – a heater membrane and a sensor membrane such that the films could be placed across the two membranes and the in-plane heat flow measurements could be performed. Each membrane is 2 μm thick, 80 μm long and 60 μm wide. They also have a platinum resistor patterned and deposited on top of them which can act both as a heater and a temperature sensor. The membranes are supported by six 200 μm long beams, with a cross-section of 3 μm by 2 μm. The thin, long beams were designed to minimize the thermal conductance of the calorimetric structures to the ambient which helps in achieving a higher heat flow resolution. Details of the fabrication is discussed below.

A low-stress SiN film with a thickness of 2 μm was first deposited on a 500 μm thick silicon (Si) wafer using LPCVD (Low Pressure Chemical Vapor Deposition) (Step 1). For the role of the heater and thermometer, a 30 nm thick platinum (Pt) serpentine film was then deposited using a physical vapor deposition (PVD) technique and patterned over the SiN layer using lithography and a lift-off process (Step 2). Using a similar process as in step 2, a 1 μm thick gold (Au) film was then patterned and deposited to extend electrical connections from the Pt serpentine (Step 3) and would be used later to connect to the external circuitry via wire bonding. The contour of the suspended membranes and the support beams is formed by RIE (Reactive Ion Etch) on the SiN layer on the front side of the wafer and a window on the SiN layer on the back side (Step 4). DRIE is performed using Bosch process to etch through the 500 μm thick Si from the back side of the wafer to suspend the designed devices, which were then cleaned and released using Acetone and IPA (Iso-Propyl Alcohol) (Step 5).

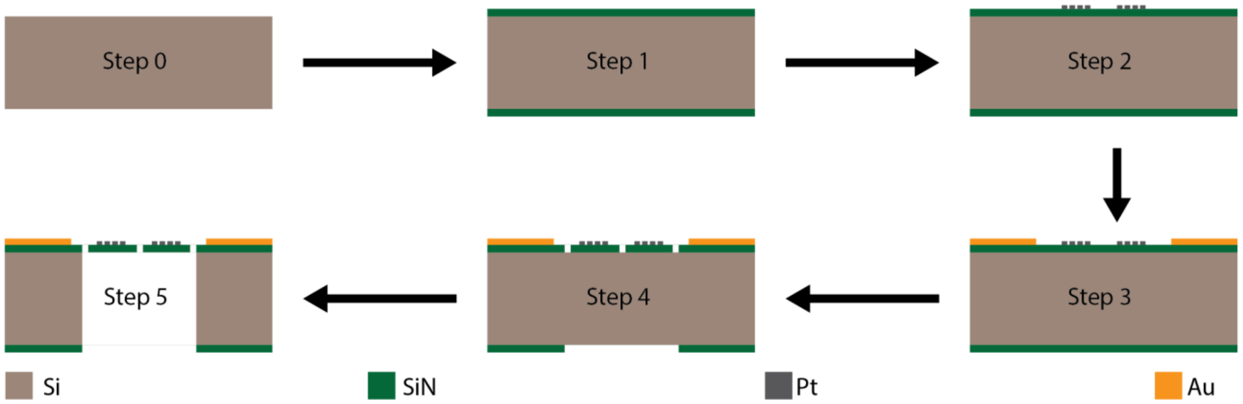

Figure S3.1 Fabrication details of SiN based suspended calorimeters used for the in-plane thermal conductivity measurements.

Once the calorimeters are fabricated, we transfer the 2DP samples onto the calorimeters carefully using a micro-manipulator under a stereo microscope. During the transfer process, the suspended membranes were dipped in Iso-propyl alcohol (IPA) and right before the surfaces are about to dry out, the film is carefully dropped on the membranes. The residual liquid bonds the film to the membranes which helps in ensuring good thermal contact. We then take this device to an SEM (Helios 650) with focused-ion beam (FIB) capabilities and carefully cut through the suspended region of the film to give it a uniform, rectangular shape which makes it trivial to estimate the thermal conductivity from a cross-conductance measurement. Extra care is taken while using the FIB to minimize the Ga^+ ion deposition and heat dissipation by using low beam powers to cut the film. Notably, we compared measurements between samples that were and were not FIB processed, which gave results consistent with sample-to-sample variance. This suggests that FIB processing does not meaningfully change the thermal conductivities we extract. Next, we deposit platinum on the ends of the film to improve its thermally contact with the suspended membranes. This helps in minimizing the effects of contact resistance during the measurement.

Next, we move the device into a cryostat (Janis ST-100) with the capability of performing measurements at low temperatures. The device chamber is pumped down to ~10^{-6} torr. The cryostat uses a combination of liquid N_2 and a PID controlled heater to set the ambient temperature at a given set point between 100-300 K. We start with a temperature coefficient of resistance (TCR) measurement of the platinum resistor by measuring the resistance at temperatures ranging from 100 K to 300 K. Following this, we measure the thermal frequency response of the suspended devices to determine the time constant of the calorimeters and the corresponding frequency dependent attenuation in the temperature rise. See next section for details about the TCR and the frequency response measurements.

Next, we perform experiments to determine the cross-conductance between the two membranes at various ambient temperatures. We start by dissipating a known amount of power (1 μW – 2.5 μW) in the heater membrane via Joule heating, by supplying an ac current of amplitude I_{ac} and a frequency (f) of 0.5 Hz. This frequency was chosen because it is much lower than the thermal roll-off frequency determined from the frequency response measurement, and thus a full thermal response is obtained. This power dissipation sets up temperature oscillations in the heater membrane at $2f$. We can estimate the amplitude of this temperature oscillation ($\Delta T_{heater,2f}$) by measuring the amplitude of voltage oscillations ($\Delta V_{heater,3f}$) at $3f$ using a lock-in technique[11]. The expression is given as:

$$\Delta T_{heater,2f} = \frac{2\Delta V_{heater,3f}}{I_{ac} R_h \alpha} \tag{3.1}$$

where R_h is the electrical resistance of the heater and α is the TCR of the platinum resistor.

This temperature oscillation on the heater then couples into the sensor membrane through the film and also sets up temperature oscillations on the sensor at $2f$. We pass a dc current of known magnitude (I_{dc}), which we have chosen to be 10 μA for all experiments, across the sensor membrane and pick up the amplitude of voltage oscillations ($\Delta V_{sensor,2f}$) at $2f$ using another lock-in instrument. We can then estimate the amplitude of the temperature oscillations ($\Delta T_{sensor,2f}$) on the sensor using the following expression:

$$\Delta T_{sensor,2f} = \frac{\Delta V_{sensor,2f}}{I_{dc} R_s \alpha} \tag{3.2}$$

where R_s is the electrical resistance of the sensor.

Once we have estimated the temperature rise on both the membranes, the in-plane conductance ($G_{||}$) across the membranes can be estimated by assuming a thermal model (S3.3) for the system. The expression for cross-conductance is given as follows:

$$G_{||} = \frac{G_{th} \times \Delta T_{sensor,2f}}{\left(\Delta T_{heater,2f} - \Delta T_{sensor,2f}\right)} \tag{3.3}$$

where G_{th} is the beam conductance of the suspended membrane to the ambient and is assumed equal for both the membranes as both the devices are identical in all dimensions.

Next, we measure the length (l), width (w) and thickness (t) of the film on a confocal microscope (Olympus OLS 4000 LEXT) which allows us to extract the conductivity (k) from cross-conductance values using the following expression:

$$k = \frac{l \cdot G_{||}}{w \cdot t} \tag{3.4}$$

3.2 Characterization of the thermal frequency response and TCR of the suspended calorimeters.

We characterized the TCR (α) of our platinum resistors in the cryostat (Janis ST-100). At each setpoint temperature (T), the resistance of a suspended calorimeter (R) was measured by passing an ac current having an amplitude of 1 μA and a frequency of 97 Hz. This current was chosen to ensure that there was no self-heating in the device. The setpoint temperature was then varied from 300 K to 100 K. Figure S3.2a shows the measurement results. We note that the resistance of platinum obtained has a small nonlinearity over the large temperature range. We account for this nonlinearity by having a 2nd order polynomial fit to determine the slope at each temperature. We can then estimate the TCR by using the following expression

$$\alpha(T) = \left(\frac{1}{R(T)}\right) \times \frac{dR}{dT} \tag{3.5}$$

The thermal frequency response of our suspended calorimeters was characterized at room temperature. We supplied a sinusoidal heating current of known amplitude (I_{ac} = 10μA) to one of the suspended devices at a frequency (f) which results in temperature oscillations at $2f$ via joule heating. We then estimate the amplitude of these oscillations (ΔT) by measuring the amplitude of voltage oscillations (ΔV) at $3f$ as follows

$$\Delta T = \frac{2\Delta V}{I_{ac} R \alpha} \tag{3.6}$$

where R is the electrical resistance and α is the TCR of the platinum resistor. The frequency is then swept from 0.05 Hz to 50 Hz and the corresponding temperature rise is measured. Figure S3.2b shows the normalized temperature rise as a function of the heating frequency. As can be seen from

the figure, a heating frequency of 1 Hz is much below the thermal roll-off frequency (~ 5 Hz) and hence all measurements have been carried out at this frequency.

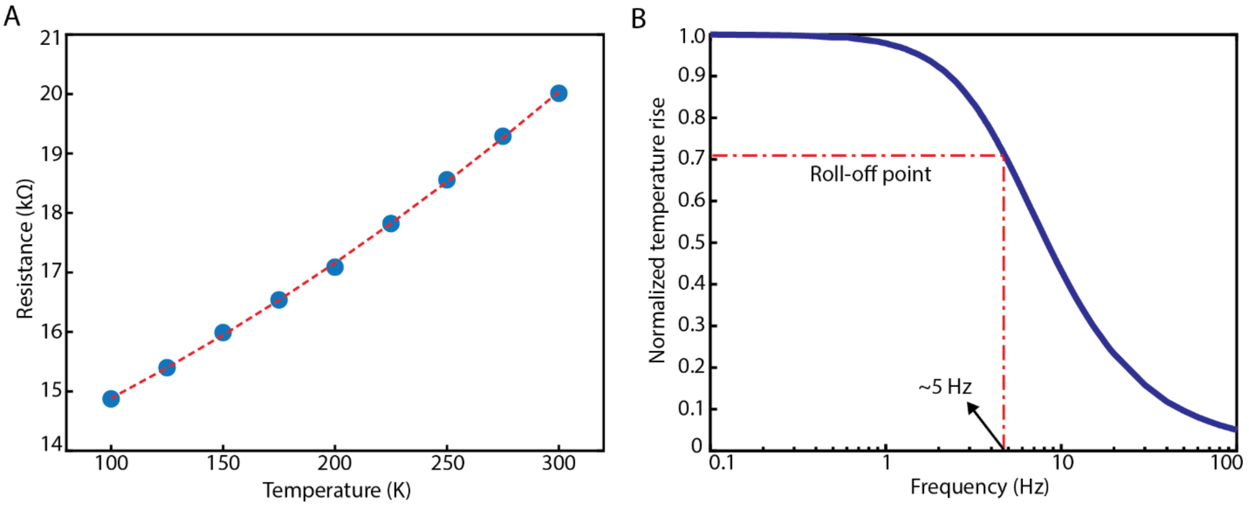

Figure S3.2 a) Resistance of the platinum resistor on a representative device vs ambient temperature. b) Thermal frequency response of a representative device at 300 K.

3.3 Description of the thermal model used for in-plane conductivity measurements.

Figure S3.3a shows the thermal model used for analyzing the in-plane measurement results. In the model, G_{th} is the structural beam conductance of one membrane and $G_{||}$ is the in-plane conductance through the 2DP sample. Due to the symmetry in the device fabrication, we assume the beam conductance to be equal for both membranes. We start the analysis by dissipating a known amount of power P_{Joule} in the heater membrane. There are two channels by which this heat flows to the ambient, which is shown as Q_1 and Q_2. The temperature rise on the heating side and sensing side is also shown in the model as ΔT_h and as ΔT_s respectively. Writing down the expressions for Q_1 and Q_2:

$$Q_1 = G_{th} \cdot \Delta T_h \tag{3.7}$$

$$Q_2 = G_{||} \cdot (\Delta T_h - \Delta T_s) = G_{th} \cdot \Delta T_s \tag{3.8}$$

From the conservation of energy, the following condition is imposed

$$P_{Joule} = Q_1 + Q_2 \tag{3.9}$$

From equations (3.7), (3.8) and (3.9) we can obtain the expression for G_{th}:

$$G_{th} = \frac{P_{Joule}}{\Delta T_h + \Delta T_s} \tag{3.10}$$

Once G_{th} is determined, we can use equation (10) to estimate

$$G_{||} = \frac{Q_2}{\Delta T_h - \Delta T_s} = \frac{G_{th} \cdot \Delta T_s}{\Delta T_h - \Delta T_s} \tag{3.11}$$

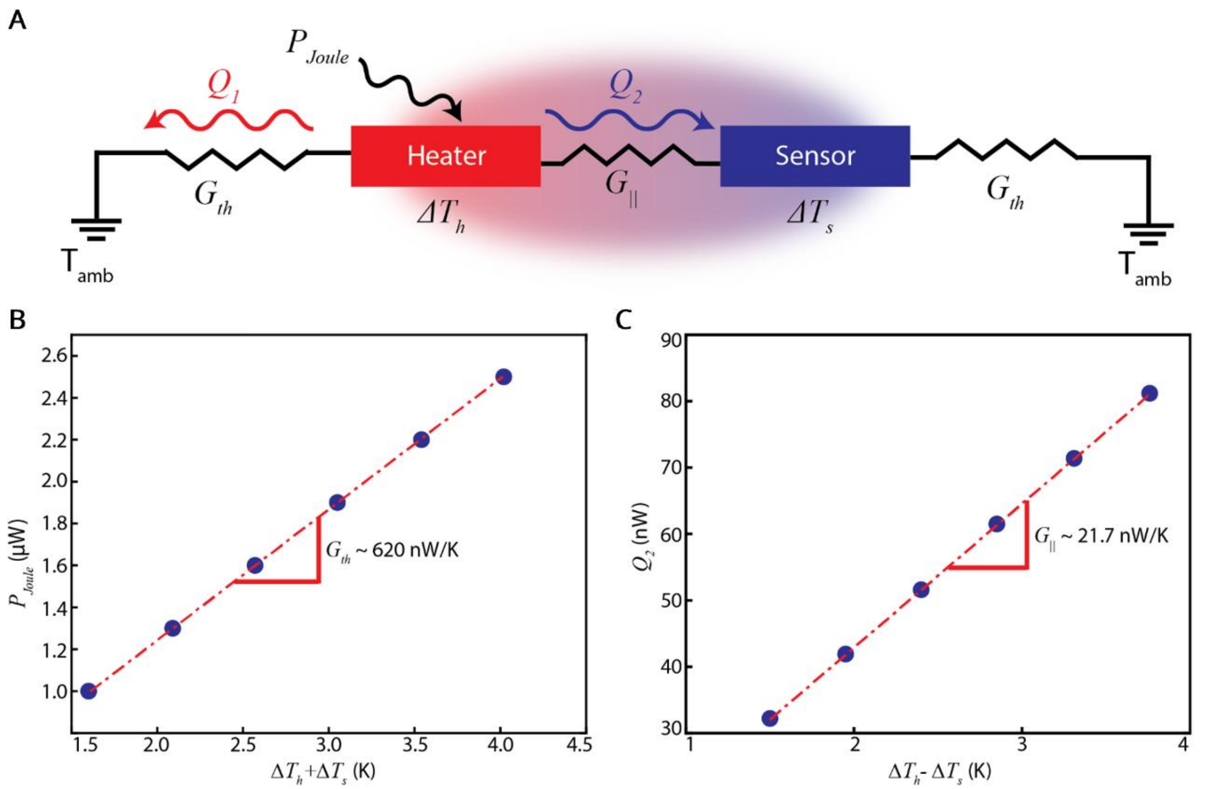

Figure S3.3 a) Illustration of the thermal model used to analyze the in-plane measurement results. b) Data for the measurement of the beam conductance on a representative sample at 300 K. c) Data for the measurement of the cross conductance on a representative sample at 300 K.

3.4 Effect of Focused Ion Beam exposure and thermal contact resistance on the measured in-plane thermal conductivity.

Figure S3.4a and S3.4b show two TAPPy-PDA films (Sample A and Sample B) transferred onto our suspended calorimeters, where the gap between the two membranes is 25 µm and 50 µm, respectively. Sample A was measured after a FIB cut to make the film more rectangular in shape, followed by Pt deposition to improve the thermal contact with the membrane substrate, whereas sample B was measured without any FIB exposure and Pt deposition. The TAPPy-PDA film on sample B was fairly rectangular right after the transfer and thus could be used to make a fair comparison with sample A. Also, the different gap size between samples A and B allowed for a length dependent measurement of the thermal conductivity, which provides more insights into the role of contact resistance on the measurement. An outline of the rectangle defining the film dimensions for our analysis is shown in both the figures.

Figure S3.4c and S3.4d show the in-plane conductance of the two suspended films. Confocal scans were done to determine the thickness of each film. Sample A was found to be 67 nm thick and sample B was found to be 56 nm thick. Using the measured in-plane conductance values, along with the film dimensions, we find the in-plane thermal conductivity at 300 K to be 0.45 W $m^{-1}K^{-1}$ and 0.47 W $m^{-1}K^{-1}$ for sample A and B respectively. This variation is well within our sample-to-sample uncertainty of 0.06 W $m^{-1}K^{-1}$ as is reported in the main manuscript. This measurement

allows us to conclude that the FIB exposure and Pt deposition play a minimal role on the physical properties of the transferred films and there seems to be negligible contact resistance after a film is successfully transferred onto the suspended membranes.

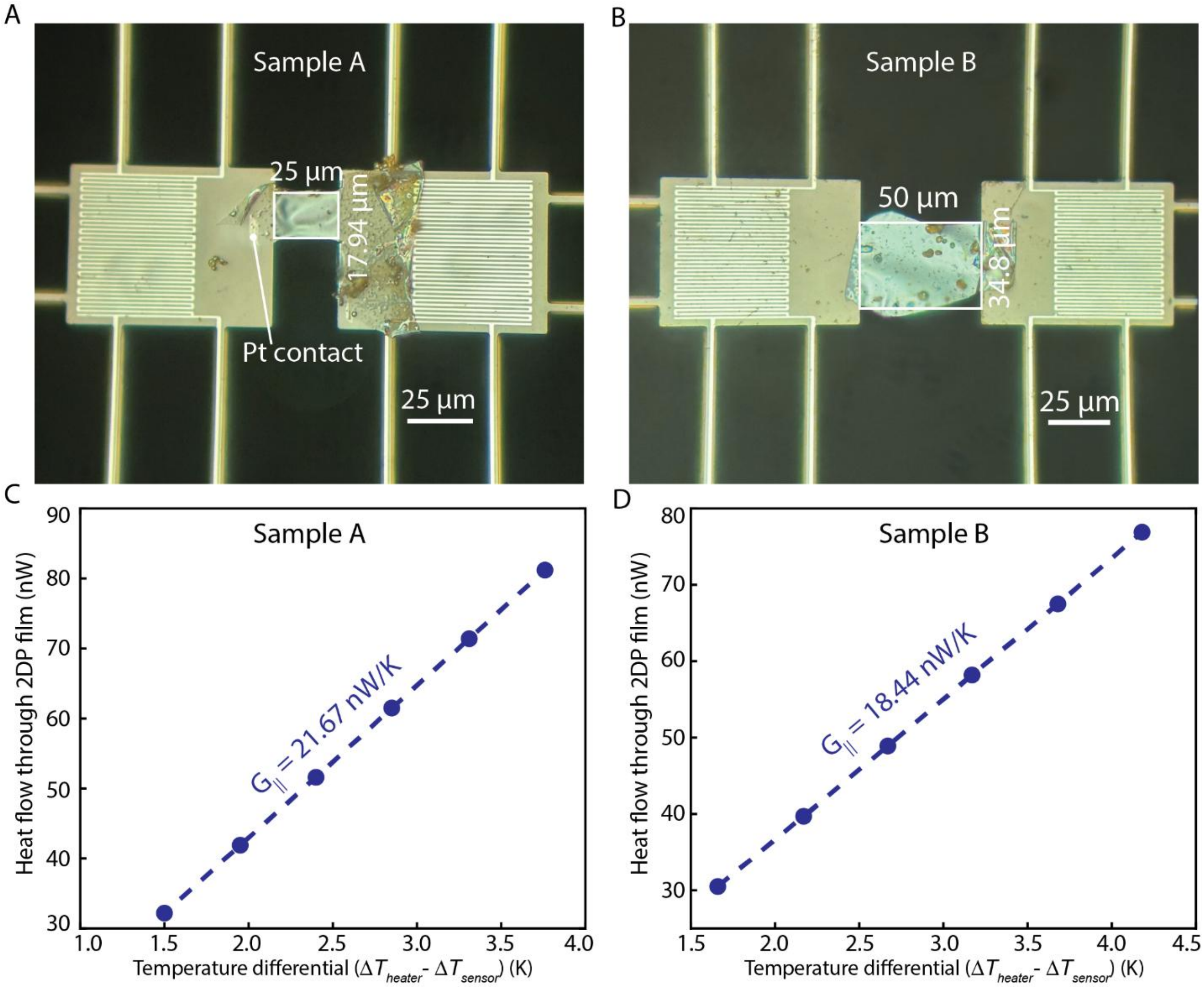

Figure S3.4. a) Light microscope image of Sample A, which comprises of a TAPPy-PDA film transferred onto our suspended calorimeters, which has then been FIB cut into a rectangular shape and has Pt deposition to improve the thermal contact. The gap size between the calorimeters is 25 µm and the width of the film is 17.94 µm. Confocal scans revealed a thickness of 67 nm for this film. b) Same as in (a), however, here, the gap size between the two suspended membranes is 50 µm, and the width of the transferred film is approximately 34.8 µm (rectangular outline to approximate the film dimensions is shown in both the figures). The thickness of this film was found to be 56 nm. c) Data corresponding to the in-plane thermal conductance measurement of sample A at 300 K. Using the film dimensions mentioned above, this conductance corresponds to an in-plane conductivity $k_{\parallel} = 0.45$ W m^{-1}K^{-1}. d) Same as in (c), but for sample B. This conductance corresponds to an in-plane conductivity $k_{\parallel} = 0.47$ W m^{-1}K^{-1}.

4 Lattice Dynamics

Lattice dynamics simulations of 1x1x10 supercells with a 2x2x1 phonon wave vector mesh for TAPPy-PDA and Per-PDA were performed using the GULP software package using the UFF force field with Coulomb interactions.[19] To this end, each supercell was initially relaxed under constant volume constraints, followed by constant pressure constraints at 1 atm. The temperature-dependent lattice parameters are listed in Table 4.1 for TAPPy-PDA and Table 4.2 for Per-PDA. The phonon mode frequencies were then used to calculate the specific heats for temperatures ranging from 77 K to 400 K (Figure S4.1), employing the Bose-Einstein statistics to describe the phonon occupations. An identical process was used to predict the heat capacity of COF-5, and a value of 0.44 J/cm^3-K at 300 K was predicted, which is within the uncertainty of its experimentally measured heat capacity of 0.52±0.08 J/cm^3-K at 300 K.[20]

Table S4.1. TAPPy-PDA: T dependent k UFF+EQeq Lattice Parameters

	77 K	100 K	150 K	200 K	250 K	300 K	350 K	400 K
a	37.28	37.24	37.14	37.03	36.92	36.78	36.50	34.98
b	31.75	31.78	31.86	31.95	32.03	32.14	32.38	33.64
c	3.98	3.99	4.03	4.07	4.11	4.15	4.21	4.30
α	90.00	90.01	90.01	90.00	90.03	89.97	89.88	89.28
β	76.39	76.30	76.12	75.92	75.67	75.39	74.92	73.29
γ	90.00	90.00	90.00	90.00	90.00	90.00	90.00	90.00

Table S4.2. Per-PDA: T dependent k UFF+EQeq Lattice Parameters

	77 K	100 K	150 K	200 K	250 K	300 K	350 K	400 K
a	34.02	34.00	33.95	33.90	33.85	33.80	33.75	33.67
b	39.57	39.58	39.60	39.63	39.65	39.67	39.69	39.74
c	3.66	3.67	3.69	3.70	3.72	3.74	3.75	3.77
α	89.99,	89.99	89.99	89.99	89.99	90. 00	90. 00	90. 00
β	70.72	70.71	70.69	70.64	70.60	70.50	70.43	70.25
γ	90.00	90.00	90.00	90.00	90.00	90.00	90.00	90.00

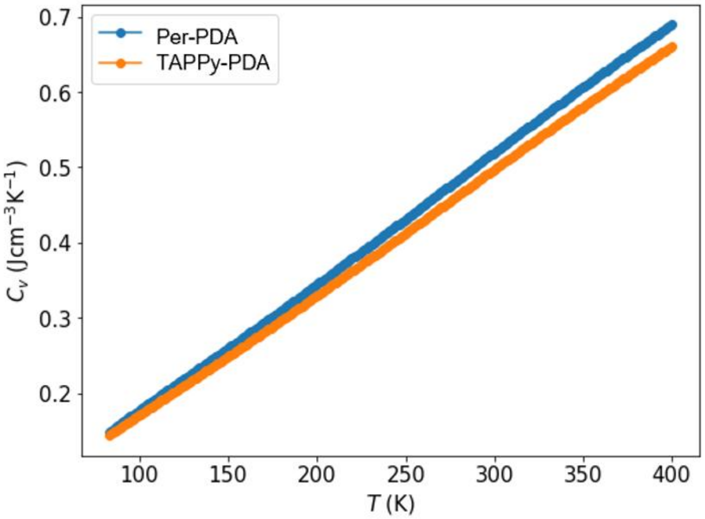

Figure S4.1: Temperature dependent volumetric heat capacity of 2DPs

5 References

(1) Oanta, A. K.; Collins, K. A.; Evans, A. M.; Pratik, S. M.; Hall, L. A.; Strauss, M. J.; Marder, S. R.; D'Alessandro, D. M.; Rajh, T.; Freedman, D. E.; Li, H.; Brédas, J.-L.; Sun, L.; Dichtel, W. R. Electronic Spin Qubit Candidates Arrayed within Layered Two-Dimensional Polymers. *J. Am. Chem. Soc.* **2023**, *145* (1), 689–696. https://doi.org/10.1021/jacs.2c11784.

(2) Ascherl, L.; Evans, E. W.; Gorman, J.; Orsborne, S.; Bessinger, D.; Bein, T.; Friend, R. H.; Auras, F. Perylene-Based Covalent Organic Frameworks for Acid Vapor Sensing. *J. Am. Chem. Soc.* **2019**, *141* (39), 15693–15699. https://doi.org/10.1021/jacs.9b08079.

(3) Feriante, C. H.; Jhulki, S.; Evans, A. M.; Dasari, R. R.; Slicker, K.; Dichtel, W. R.; Marder, S. R. Rapid Synthesis of High Surface Area Imine-Linked 2D Covalent Organic Frameworks by Avoiding Pore Collapse During Isolation. *Advanced Materials* **2020**, *32* (2), 1905776. https://doi.org/10.1002/adma.201905776.

(4) Matsumoto, M.; Valentino, L.; Stiehl, G. M.; Balch, H. B.; Corcos, A. R.; Wang, F.; Ralph, D. C.; Mariñas, B. J.; Dichtel, W. R. Lewis-Acid-Catalyzed Interfacial Polymerization of Covalent Organic Framework Films. *Chem* **2018**, *4* (2), 308–317. https://doi.org/10.1016/j.chempr.2017.12.011.

(5) Schmidt, A. J.; Cheaito, R.; Chiesa, M. A Frequency-Domain Thermoreflectance Method for the Characterization of Thermal Properties. *Review of Scientific Instruments* **2009**, *80* (9), 094901. https://doi.org/10.1063/1.3212673.

(6) Regner, K. T.; Majumdar, S.; Malen, J. A. Instrumentation of Broadband Frequency Domain Thermoreflectance for Measuring Thermal Conductivity Accumulation Functions. *Review of Scientific Instruments* **2013**, *84* (6), 064901. https://doi.org/10.1063/1.4808055.

(7) Cahill, D. G. Analysis of Heat Flow in Layered Structures for Time-Domain Thermoreflectance. *Review of Scientific Instruments* **2004**, *75* (12), 5119–5122. https://doi.org/10.1063/1.1819431.

(8) Malen, J. A.; Baheti, K.; Tong, T.; Zhao, Y.; Hudgings, J. A.; Majumdar, A. Optical Measurement of Thermal Conductivity Using Fiber Aligned Frequency Domain Thermoreflectance. *Journal of Heat Transfer* **2011**, *133* (8). https://doi.org/10.1115/1.4003545.

(9) Schmidt, J. A.; Cheaito, R.; Chiesa, M.; Schmidt, A. J.; Cheaito, R.; Chiesa, M. A Frequency-Domain Thermoreflectance Method for the Characterization of Thermal Properties. *Review of Scientific Instruments* **2009**, *80* (9), 94901. https://doi.org/10.1063/1.3212673.

(10) Epstein, J.; Ong, W. L.; Bettinger, C. J.; Malen, J. A. Temperature Dependent Thermal Conductivity and Thermal Interface Resistance of Pentacene Thin Films with Varying Morphology. *ACS Applied Materials and Interfaces* **2016**, *8* (29), 19168–19174. https://doi.org/10.1021/acsami.6b06338.

(11) Sadat, S.; Meyhofer, E.; Reddy, P. High Resolution Resistive Thermometry for Micro/Nanoscale Measurements. *Review of Scientific Instruments* **2012**, *83* (8), 084902. https://doi.org/10.1063/1.4744963.

(12) Panda, K.; Mittapally, R.; Reddy, P.; Yadlapalli, S.; Meyhofer, E. Micro-Kelvin Temperature-Stable System for Biocalorimetry Applications. *Review of Scientific Instruments* **2024**, *95* (3), 034902. https://doi.org/10.1063/5.0188285.

(13) Sadat, S.; Meyhofer, E.; Reddy, P. Resistance Thermometry-Based Picowatt-Resolution Heat-Flow Calorimeter. *Applied Physics Letters* **2013**, *102* (16), 163110.

https://doi.org/10.1063/1.4802239.
(14) Zheng, J.; Wingert, M. C.; Dechaumphai, E.; Chen, R. Sub-Picowatt/Kelvin Resistive Thermometry for Probing Nanoscale Thermal Transport. *Review of Scientific Instruments* **2013**, *84* (11), 114901. https://doi.org/10.1063/1.4826493.
(15) Yang, L.; Yue, S.; Tao, Y.; Qiao, S.; Li, H.; Dai, Z.; Song, B.; Chen, Y.; Du, J.; Li, D.; Gao, P. Suppressed Thermal Transport in Silicon Nanoribbons by Inhomogeneous Strain. *Nature* **2024**, *629* (8014), 1021–1026. https://doi.org/10.1038/s41586-024-07390-4.
(16) Luan, Y.; Yan, S.; Panda, K.; Majumder, A.; Guan, J.; Mittapally, R.; Meyhofer, E.; Reddy, P. The Metal–Insulator Transition in Vanadium Oxide Nanofilms Enables Microkelvin-Resolution Thermometry. *Nano Lett.* **2024**, *24* (23), 7048–7054. https://doi.org/10.1021/acs.nanolett.4c01535.
(17) Majumder, A.; Thompson, D.; Mittapally, R.; Reddy, P.; Meyhofer, E. Quantifying the Spatial Distribution of Radiative Heat Transfer in Subwavelength Planar Nanostructures. *ACS Photonics* **2023**, acsphotonics.2c01901. https://doi.org/10.1021/acsphotonics.2c01901.
(18) Thompson, D.; Zhu, L.; Mittapally, R.; Sadat, S.; Xing, Z.; McArdle, P.; Qazilbash, M. M.; Reddy, P.; Meyhofer, E. Hundred-Fold Enhancement in Far-Field Radiative Heat Transfer over the Blackbody Limit. *Nature* **2018**, *561* (7722), 216–221. https://doi.org/10.1038/s41586-018-0480-9.
(19) Gale, J. D.; Rohl, A. L. The General Utility Lattice Program (GULP). *Molecular Simulation* **2003**, *29* (5), 291–341.
(20) Evans, A. M.; Giri, A.; Sangwan, V. K.; Xun, S.; Bartnof, M.; Torres-Castanedo, C. G.; Balch, H. B.; Rahn, M. S.; Bradshaw, N. P.; Vitaku, E.; Burke, D. W.; Li, H.; Bedzyk, M. J.; Wang, F.; Brédas, J.-L.; Malen, J. A.; McGaughey, A. J. H.; Hersam, M. C.; Dichtel, W. R.; Hopkins, P. E. Thermally Conductive Ultra-Low-k Dielectric Layers Based on Two-Dimensional Covalent Organic Frameworks. *Nat. Mater.* **2021**, *20* (8), 1142–1148. https://doi.org/10.1038/s41563-021-00934-3.
